# Kinome capture sequencing of high-grade serous ovarian carcinoma reveals novel mutations in the *JAK3* gene

**DOI:** 10.1371/journal.pone.0235766

**Published:** 2020-07-08

**Authors:** Lorenza Mittempergher, Anna M. Piskorz, Astrid J. Bosma, Magali Michaut, G. Bea A. Wisman, Roelof J. C. Kluin, Marja Nieuwland, Wim Brugman, Kevin J. W. van der Ven, Francesco Marass, James Morris, Nitzan Rosenfeld, Mercedes Jimenez-Linan, Steven de Jong, Ate G. J. van der Zee, James D. Brenton, René Bernards

**Affiliations:** 1 Division of Molecular Carcinogenesis, Oncode Institute, The Netherlands Cancer Institute, Amsterdam, The Netherlands; 2 Cancer Research UK Cambridge Institute University of Cambridge, Li Ka Shing Centre, Cambridge, United Kingdom; 3 Department of Gynecologic Oncology, Cancer Research Center Groningen, University of Groningen, Groningen, The Netherlands; 4 Genomics Core Facility, The Netherlands Cancer Institute, Amsterdam, The Netherlands; 5 Department of Pathology, University Medical Centre Utrecht, Utrecht, The Netherlands; 6 Department of Biosystems Science and Engineering and Swiss Institute of Bioinformatics, ETH Zurich, Basel, Switzerland; 7 Cancer Research UK Major Centre–Cambridge, Cancer Research UK Cambridge Institute, Cambridge, United Kingdom; 8 Cambridge University Hospitals NHS Foundation Trust, Cambridge, United Kingdom; 9 Department of Medical Oncology, Cancer Research Center Groningen, University of Groningen, University Medical Center Groningen, Groningen, The Netherlands; University of Hawaii System, UNITED STATES

## Abstract

High-grade serous ovarian carcinoma (HGSOC) remains the deadliest form of epithelial ovarian cancer and despite major efforts little improvement in overall survival has been achieved. Identification of recurring “driver” genetic lesions has the potential to enable design of novel therapies for cancer. Here, we report on a study to find such new therapeutic targets for HGSOC using exome-capture sequencing approach targeting all kinase genes in 127 patient samples. Consistent with previous reports, the most frequently mutated gene was *TP53* (97% mutation frequency) followed by *BRCA1* (10% mutation frequency). The average mutation frequency of the kinase genes mutated from our panel was 1.5%. Intriguingly, after *BRCA1*, *JAK3* was the most frequently mutated gene (4% mutation frequency). We tested the transforming properties of JAK3 mutants using the Ba/F3 cell-based *in vitro* functional assay and identified a novel gain-of-function mutation in the kinase domain of *JAK3* (p.T1022I). Importantly, p.T1022I *JAK3* mutants displayed higher sensitivity to the JAK3-selective inhibitor Tofacitinib compared to controls. For independent validation, we re-sequenced the entire *JAK3* coding sequence using tagged amplicon sequencing (TAm-Seq) in 463 HGSOCs resulting in an overall somatic mutation frequency of 1%. TAm-Seq screening of *CDK12* in the same population revealed a 7% mutation frequency.

Our data confirms that the frequency of mutations in kinase genes in HGSOC is low and provides accurate estimates for the frequency of *JAK3* and *CDK12* mutations in a large well characterized cohort. Although p.T1022I *JAK3* mutations are rare, our functional validation shows that if detected they should be considered as potentially actionable for therapy. The observation of *CDK12* mutations in 7% of HGSOC cases provides a strong rationale for routine somatic testing, although more functional and clinical characterization is required to understand which nonsynonymous mutations alterations are associated with homologous recombination deficiency.

## Background

Epithelial ovarian cancer is the most deadly gynecological cancer in western countries accounting for almost two-thirds of ovarian cancer deaths [1,2]. The cancer is usually diagnosed at an advanced stage because of early metastatic spread from the fallopian tube [3]. High-grade serous ovarian carcinoma (HGSOC) represents 60%-80% of epithelial ovarian cancers and most deaths are associated with this subtype. Currently, the standard therapy is a combination of maximal cytoreductive surgery with platinum-taxane chemotherapy in either a neo- or adjuvant setting [3,4]. HGSOC is associated with initial chemotherapy responsiveness, however most cancers relapse and become increasingly chemotherapy resistant, with an overall 5-year survival probability of approximately 30% [4,5].

The lack of successful treatment strategies led researchers in recent years to identify genetic abnormalities in HGSOC samples with the aim to exploit these as therapeutic targets. To date, several studies have addressed this issue using a comprehensive genomic approach [2,4,6–9].

Exome sequencing studies revealed a narrow spectrum of somatic mutations in HGSOC, where *TP53* is mutated in more than 97% of the tumors and the next most frequently affected genes are mutated in less than 10% of the tumors [4,7,10]. HGSOC is strongly characterized by chromosomal instability with a large burden of copy number gains and losses [9,11,12]. Defects in homologous recombination (HR) repair occur in up to 50–60% of cases, including germline or somatic loss-of-function mutations of *BRCA1* or *BRCA2* (10%-20%), epigenetic silencing of *BRCA1* through methylation and rarer defects in other HR genes. Tumors with defective HR such as *BRCA1/2* mutated HGSOC, display particular sensitivity to PARP (Poly-ADP-Ribose Polymerase) inhibitor (PARPi), a novel class of anticancer agents, which exhibit synthetic lethality in tumors with a defective HR pathway [13–15]. *BRCA1/2* mutation status is a potent predictor of response to PARPi in this group of tumors and predicts longer survival than other causes of HRD [16–18]. However, with the exception of *BRCA1/2* mutations, little is known about other mutations in HGSOC that could become valuable targets for personalized therapy. The exome sequencing used by the TCGA consortium had a limited depth (estimated 3–30 fold), that only allows the robust identification of mutations present at high allele frequency and may have missed low abundance alleles. Other recent studies are characterized by relatively small patient groups (N <100), which limits the identification of significantly mutated genes to those with an expected frequency ≤6% [7]. Therefore, we initiated this study with the goal of identifying new therapeutic targets for HGSOC by kinome-captured next-generation sequencing in a well characterized HGSOC cohort. We deep sequenced 549 kinase genes and 48 additional cancer-related genes using a targeted exome-capture enrichment strategy. We focused on the family of kinases because these enzymes are effective targets for therapy and many kinase inhibitory drugs are already clinically validated.

## Material and methods

### Patient material and clinical data

Our discovery cohort includes 153 clinically annotated stage II-IV high-grade serous ovarian carcinoma (HGSOC) samples and 54 matched normal samples. Patient material was available from frozen tissue blocks. Matched normal DNA samples were obtained from adjacent normal tissue, peripheral lymphocytes or previously extracted germline DNA. Samples were collected at the University Medical Center Groningen (UMCG, Netherlands) (N = 77 tumor samples) and at the Cancer Research UK Cambridge Institute University of Cambridge (N = 76 tumor samples and N = 54 matched normal samples). Sections of 3μm thickness from the samples were used for hematoxylin and eosin staining and assessed by a pathologist for tumor cellularity. Specimens with tumor cell content below 10% were excluded from the mutation analysis (N = 26) ending up with 127 tumor samples and 54 matched normal DNAs. Patient characteristics are reported in the S1 Table.

Our validation cohort includes 500 formalin-fixed, paraffin-embedded (FFPE) tissue samples corresponding to 463 patients selected from the CTCR-OV04 [19], SEARCH study [20,21] and ICON7 [22] clinical studies.

For patient samples collected in the UMCG, all clinicopathological data and follow-up data was registered in an anonymous password, protected database, in compliance with Dutch law. All patients gave informed consent and the UMCG ethical review board approved the study. Data were fully anonymized prior analysis by the authors. The CTCR-OV04 study was approved by the Cambridgeshire Research Ethics Committee (reference 08/H0306/61) and all participants provided written informed consent. SEARCH was approved by the Cambridgeshire 4 Research Ethics Committee. All participants provided written informed consent. ICON7 translational research samples were provided following peer review and approval by the translational research subgroup and trial steering committee. All patients provided written informed consent. Only ICON7 patients providing consent for translational research sample collection were available for this analysis.

The study here described was performed in accordance with the Declaration of Helsinki.

### Sample processing and library construction

DNA from the Cambridge samples was isolated from the tissue using the AllPrep DNA/RNA Mini kit (Qiagen, Germany) according to manufacturer’s instruction with following changes: tissue disruption was performed using homogenization method (Precellys®24 homogenizer) and final DNA elution volume was 100μl for solid tumor tissue and 60μl for ascites samples. DNA of the Groningen samples was isolated by standard overnight Proteinase K treatment, salt-chloroform extraction, and isopropanol precipitation [23]. DNA quality was assessed following the BIOMED-2 protocol [24]. The DNA concentration was measured using the NanoDrop™ 2000 (Thermo scientific, U.S.A.) or Qubit® 2.0 Fluorometer (Life Technologies, U.S.A.). DNA samples (1.4μg total input DNA) were sheared with the S220 Focused-ultrasonicator (Covaris Inc., U.S.A.) to an average size of 150 base pair (bp). DNA fragment libraries were prepared with the TruSeq DNA Sample Preparation Kit (Illumina, U.S.A.). Briefly, the DNA fragments were end repaired and A tailed, then indexed Illumina compatible (P5/P7) linkers were added by ligation and the fragments are enriched by PCR. Libraries were amplified with 6 cycles of PCR, which yielded 450‐500ng of library. Target enrichment was performed using the human kinome DNA capture baits (Agilent Technologies, U.S.A.). The SureSelect Human Kinome 3.2Mb bait set targets 549 kinase genes and 48 additional cancer-related genes and the associated Untranslated Regions (UTRs) (S2A Table). The Agilent SureSelect Target Enrichment system, was further optimized in our laboratory [25,26] and uses biotinylated RNA probes to selectively capture kinase genes prior to sequencing. The size and concentration of captured DNA was measured using a 2100 Bioanalyzer (Agilent Technologies, U.S.A.). Six libraries were pooled for each capture reaction, with 100ng of each library, and the following custom blockers were added to prevent hybridization to adapter sequences: B1:5’AGATCGGAAGAGCACACGTCTGAACTCCAGTCACNNNNNNATCTCGTATGCCGTCTTCTGCTTG/3’ddC; B2: 5’CAAGCAGAAGACGGCATACGAGATNNNNNNGTGACTGGAGTTCAGACGTGTGCTCTTCCGATCT/3’ddC.

### High-throughput kinome sequencing

Captured DNA samples were sequenced on a HiSeq 2000 instrument (Illumina, U.S.A) with a paired-end 51bp protocol. A total of 12 DNA libraries (pooling two captures) at 10nM concentration were sequenced per lane. Sequence reads were aligned to the human genome (GRCh37/hg19) using Burrows-Wheeler Aligner (BWA [27]) version 0.5.10 and unique pairs were used for variant calling.

### Somatic mutation calling

Candidate variants were identified using SAMtools [28] and the following inclusion criteria were applied: minimum variant frequency equal to 10%, minimum coverage equal to 10, minimum variant count equal to 5, a variant must be detected on both strands (specifically 5% of the total number of reads in each direction must have the alternative allele/variant). Variants were assessed using the Ensembl variant effect predictor (v62) [29] to define those that were likely to impact protein coding sequence (e.g. non-synonymous variants) and to filter out germline polymorphisms. Specifically, we applied a protein-altering filter, which only selects variants classified as missense, nonsense, splice site and insertions or deletions classified as frame shifting. An additional 284 normal samples available in our laboratory at the NKI-AVL institute (including the 54 matched normal samples mentioned above), were used to improve the removal of germline SNPs and sequencing errors applying the following rules: a) all variants present in more than 3 normal samples were considered germline; b) all variants present in dbSNP (http://www.ncbi.nlm.nih.gov/SNP/) or HGMD (http://www.hgmd.org/) or EVS (http://evs.gs.washington.edu/EVS/) databases and not in COSMIC (v69) (http://cancer.sanger.ac.uk/cancergenome/projects/cosmic/) database were considered germline.

In order to identify significantly mutated genes in our cohort, we employed a binomial test that determines whether the number of mutations occurring in a gene is larger than expected by chance, i.e. predicted by the Binomial null model. A binomial test assumes that mutations are independent and thereby randomly distributed along the genome. Correction for multiple testing was performed using the Benjamini Hochberg False Discovery Rate (FDR) calculation. Analyses were performed using the statistical language R (http://www.r-project.org/).

### Variant validation

*TP53* gene was sequenced using both capillary (Big Dye Terminator V3.1 Sequencing Kit, Applied Biosystems, Life Technologies, U.S.A.) and Tagged Amplicon Sequencing (TAm-Seq) as previously described [30,31]. Briefly, capillary sequencing of coding *TP53* exons 2–11 was performed using primers that encompassed the entire exon and exon–intron boundaries. Purified PCR products were sequenced using an ABI 3100 genetic analyzer (Applied Biosystems, Life Technologies, U.S.A.). Sequencing reactions were performed in forward and reverse directions. Mutational analysis was performed using SeqScape Software v2.6 (Applied Biosystems, Life Technologies, U.S.A.).

*JAK3* variants were verified using capillary sequencing on both tumor and normal matched tissue. If matched normal tissue was not available, Laser Capture Micro dissection (LCM) was performed on frozen tumor slices of 10μm (MembraneSlide 1.0 PEN, Zeiss, U.K.) in order to isolate normal cells. Briefly, the slices were stained for 10 seconds with Mayer's hemalum solution (Merck Millipore, U.S.A.) and water. After drying on a heat plate (50°C) for 10 minutes, 2,5mm^2^ normal tissue was selected and dissected using the Zeiss PALM Microbeam III System (PALM, Zeiss, Germany), and then captured with PALM AdhesiveCaps 500 clear (PALM, Zeiss, Germany) with use of the PALM Robo version 3.0 software. DNA isolation was performed with the QIAGEN® DNeasy Blood and Tissue kit (Qiagen, Germany).

For validation from FFPE tissues, the coding sequence of *JAK3* and *CDK12* was sequenced using TAm-Seq as previously described [31,32]. Briefly, all prepared libraries were pooled in equimolar concentration and quantified using the DNA 1000 kit on a Bioanalyzer 2500 instrument (Agilent Technologies, U.S.A). TAm-Seq was performed using the Fluidigm 48.48 Access Array (Fluidigm, U.S.A.) followed by deep sequencing on a MiSeq instrument (Illumina, U.S.A.) using a 125bp paired-end protocol as described previously [29]. The data analysis was performed as described earlier [32]. All variants were confirmed by visual inspection using IGV software v2.3 [33]. The mutation maps were generated using the cBioPortal MutationMapper tool [34,35].

### RT-qPCR

RNA isolation from fresh frozen tumor samples was performed with the Qiagen^®^ AllPrep DNA/RNA Mini Kit (Qiagen, Germany) (specifically for samples PT-111, PT-095, PT-151, PT-130, PT-102, PT-101, PT-109, PT-123), with cesium chloride density gradient ultracentrifugation (Roche, Switzerland) followed by DNase treatment according manufacturer's protocol (MEGAscript™ T7 Transcription Kit, Ambion, Thermo-Fisher Scientific, U.S.A.) (specifically for samples PT-049, PT-024, PT-020) or with TRIzol® Reagent (Thermo-Fisher Scientific U.S.A.) (specifically for sample PT-070).

RT-qPCR assays were carried out to measure mRNA levels of genes using the 7500 Fast Real-Time PCR System (Applied Biosystems, Life Technologies, U.S.A.) as previously described [36]. cDNA for the RT-qPCR reaction was synthetized using the Maxima First Strand cDNA Synthesis Kit (Thermo Scientific, U.S.A.) and oligo(dT) primers starting from 1 μg Total RNA. Relative mRNA levels of each gene were normalized to the expression of the housekeeping genes *GAPDH* and *18S*. The SYBR Green master mix (Roche, Switzerland) were used for the RT-qPCR reaction. The 2^-ΔΔCT^ method [37] was applied to calculate the fold change expression with respect to both housekeeping genes, *GAPDH* and *18S*. Following primers were used: *GAPDH* Forward, AAGGTGAAGGTCGGAGTCAA; *GAPDH* Reverse, AATGAAGGGGTCATTGATGG; *PIM1* Forward, TCCACCGCGACATCAAGGAC; *PIM1* Reverse, ACTCTGGAGGGCTATACACTC; *PIM2* Forward, TCGAGGCCGAGTATCGACT; *PIM2* Reverse, ATTCCGGGGAATCACTTTG; *PIM3* Forward, CTGCTCAAGGACACGGTCTACAC; *PIM3* Reverse CCCCACACACCATATCGTAGAGA; *JAK3* Forward, CTGGGCAAGGGCAACTTT; *JAK3* Reverse, AGTCCCTCTGCTGGTCTGG.

### Expression plasmids

The pMSCV-JAK3 wild type vector was used as template for generation of the *JAK3* mutations pT1022I and pG888A by site-directed mutagenesis (QuikChangeTM II XL, Agilent Technologies, U.S.A.). The *JAK3* mutation pL1091P was generated by cloning a double-stranded, sequence-verified genomic block of 650bp (gBlocks Gene Fragments, Integrated DNA Technologies, U.S.A.) containing the mutation into the pMSCV-JAK3 wild type vector. pBabe-Puro-BRAF-V600E (pBP-BRAF-V600E), pBabe-Puro-Myr-AKT (pBP-Myr-AKT) and pBabe-Puro-MEK-DD (pBP-MEK-DD) vectors were used as positive controls. All the plasmid sequences were verified by capillary sequencing.

The Murine Stem Cell virus (pMSCV)–green fluorescent protein (GFP) vectors (pMSCV-empty-GFP, pMSCV-GFP-JAK3 wild type) were a kind gift of Sandrine Degryse from the Jan Cools lab.

### Cell culture, retroviral transductions and mutant selection

Ba/F3 cells were cultured in RPMI 1640 with 10% fetal calf serum (FCS) and IL3 (10 ng/μl; Peprotech) and 1% penicillin (10,000 units/mL) and 1% streptomycin (10,000 μg/mL). Phoenix-AMPHO cells were cultured in Dulbecco’s modified Eagle medium with 10% FCS, 2mM L-Glutamine and penicillin (10,000 units/mL) and 1% streptomycin (10,000 μg/mL). All cell lines were cultured at 37°C and 5% CO2.

The Ba/F3 cells were transduced by retroviral vectors encoding either *JAK3* wild-type or *JAK3* mutants that co-express GFP. pMSCV-empty, pMSCV-JAK3 wild type, pMSCV-JAK3 mutant retrovirus was produced by transfection of Phoenix-AMPHO cells using linear polyethylenimine (PEI, 25K) (Polysciences, Germany). Retrovirus containing supernatant was used to infect Ba/F3 cells. Polybrene (5 μg/mL) was added prior to infection. pMSCV vector infected cells were FACS sorted (BD FACSDiva 8.0.1) based on their GFP fluorescent signal; pBP vector infected cells were selected by 1μg/ml Puromycin.

Ba/F3 cells were a kind gift of the Prof. Paul Coffer laboratory (Regenerative Medicine Center, UMC Utrecht).

### Protein lysate preparation and Western blotting

Cells were lysed in RIPA buffer containing 150mM NaCl, 50mM Tris pH 8.0, 1% NP-40, 0.5% sodium deoxycholate and 0.1% SDS supplemented with protease inhibitors (Complete, Roche, Switzerland) and phosphatase inhibitor cocktails II and III (Sigma-Aldrich, U.S.A.). 10X reducing agent and 4X LDS sample buffer (NuPAGE™, Thermo-Fisher Scientific, U.S.A.) were added. Subsequently, the samples were boiled for 5 minutes and quickly centrifuged at 21,000 rcf. Proteins were quantified using the BCA Protein Assay Kit (Pierce, Thermo-Fisher Scientific, U.S.A.). Equal amounts of protein were subjected to SDS gel electrophoresis using 4–12% Bis-Tris Plus Bolt gels (Thermo-Fisher Scientific, U.S.A.) and 3-(N-morpholino) propanesulfonic acid (MOPS) buffer, followed by Western blotting. Western blotting was performed using antibodies from Cell Signaling (MA, U.S.A.) against total JAK3 (3775S), phospho-JAK3 (Tyr980/981, 5031S), STAT5 (9363S), phospho-STAT5 (Tyr694, 9351S), p44/42 MAPK (Erk1/2, 137F5, 4695S), P-p44/p42 MAPK (T202/Y204, 4370S), AKT (pan, C67E7, 4691L), phospho-AKT (S473, D9E, 4060L), GAPDH and antibody from Santa Cruz Biotechnology Inc. (U.S.A.) against HSP90 α/β (f-8, SC-13119). For the western blotting results representative examples of at least two independent experiments are reported.

### Cytokine withdrawal assay and drug response assay

Cell proliferation was assessed using the IncuCyte® ZOOM System (EssenceBioscience, U.S.A.) following manufacturer's instructions. Briefly, Ba/F3 cells were washed twice with PBS in order to remove Interleukin 3 (IL-3) and resuspended in AimV medium (fully-defined serum-free media, Thermo-Fisher Scientific, U.S.A.). Afterwards cells were cultured in AimV medium for 12 hours in order to synchronize them to a growth restriction-induced quiescence [38]. Subsequently, cells were resuspended in 10% FCS RPMI medium with and without IL3 in a 96-well plate format and followed for 13 days with the IncuCyte® ZOOM System.

Ba/F3 cell lines were treated with increasing concentration of JAK1/JAK3 inhibitor Tofacitinib (CP-690550, Citrate, Selleckchem, U.S.A.) starting from 0.02μM up to 8.19μM concentration and followed for 72 hours. Following 72-hour incubation, cells were stained with CellTiter-Blue (Promega, U.S.A.) (1:30 dilution) for 4 hours and the signal measured using an EnVision multimode spectrophotometer (Perkin Elmer, U.S.A.). Each assay was carried out in biological triplicate. Each replicate of a dose-response experiment was further analyzed to calculate the IC_50_ (dose at which viability is 50% of the untreated control).

## Results

### Mutation analysis confirms TP53 and BRCA1 as most frequently mutated genes in the HGSOC dataset

We collected a discovery cohort of frozen tissue blocks from 153 clinically annotated stage II-IV HGSOC samples and 54 matched normal samples from two clinical sites (Cambridge, United Kingdom and Groningen, The Netherlands). From these, 127 (83%) samples met the inclusion criteria of greater than 10% tumor DNA. Clinicopathological characteristics of the study patient population are reported in S1 Table. On average we generated 35 million reads for each sample, of which an average of 28 million reads were unique and included for variant calling. Around 96% of the target interval had at least a 30-fold coverage and 50% had more than 150x fold coverage, which provides a high degree of sensitivity for detecting variants (S1 Fig). In the selected discovery sample set (N = 127) we observed an average of 2.4 somatic variant per Megabase (Mb) of coding sequences, based on the kinome bait set size of 3.2Mb (S2 Fig). The average number of candidate somatic mutated genes per sample was equal to 7.5 corresponding to an average of 1.2% of the genes from the kinome panel (S3 Fig, fraction of mutated genes = 0.012).

**Fig 1:**
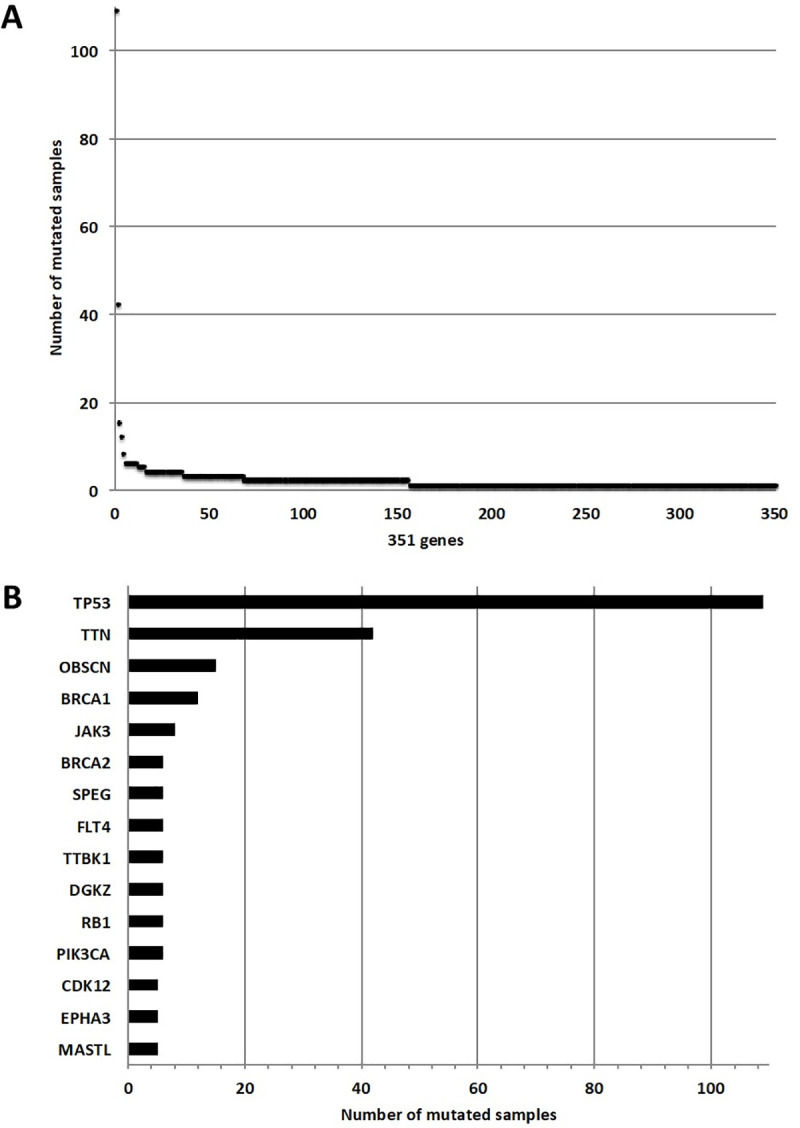
Genes mutated in the discovery ovarian dataset (N = 127). **A.** Number of samples mutated per gene: the x-axis reports the number of genes selected with the binomial test (N = 351) and the y-axis the number of samples that are mutated for each of the 351 selected genes. **B.** Top 15 mutated genes in the ovarian dataset: the x-axis reports the number of mutated samples and the y-axis the mutated genes.

A total of 3442 variants was identified with SAMtools and passed the sequencing quality filter (see Methods for details). After applying filtering to exclude SNPs, germline mutations and sequencing artifacts, we identified 798 candidate somatic variants in 351 genes, of which 321 were identified as kinase genes from the kinome targeted panel (N = 321/549 kinases; for detailed annotation see S2B Table). Of these variants, 135 (17%) were known COSMIC mutations, 14 (2%) were previously annotated in the EVS database and the remaining 649 were novel. The large majority of the 798 variants were found in the 321 kinase genes (N = 641/798, 80%). 123/321 mutated kinase genes had at least one candidate somatic variant in their kinase domain(s) (for detailed annotation see S2C Table). Out of the 127 patients, 117 (92%) had ≥1 candidate somatic variant in a kinase gene. As expected, the most frequently mutated gene was *TP53* (N = 109/127 cases, 86%) (Fig 1), although this was lower than reported in previous studies [4,8,39]. The *TP53* mutation frequency was revised to 94% (N = 120/127) after manually identifying mutations in unfiltered sequencing data (N = 8) and resequencing of 10 cases using TAm-seq (N = 3) (S3 Table, S4 Fig).

Seven samples were confirmed to be negative for *TP53* mutation. To further investigate this, we performed a second pathology review of the 7 mutation-negative cases, which resulted in the re-classification of 2 cases as low-grade serous ovarian carcinoma (LGSOC) and 1 case as mucinous ovarian carcinoma (S5 Fig for the inclusion patient diagram). The original diagnosis of HGSOC was confirmed in the remaining 4 cases. Detailed description of the mutations found in *TP53* gene can be found in Supplementary File 1.

In summary, the adjusted *TP53* mutation rate for the pathologically confirmed HGSOC cases in our set was 97% (N = 120/124).

**Fig 2:**
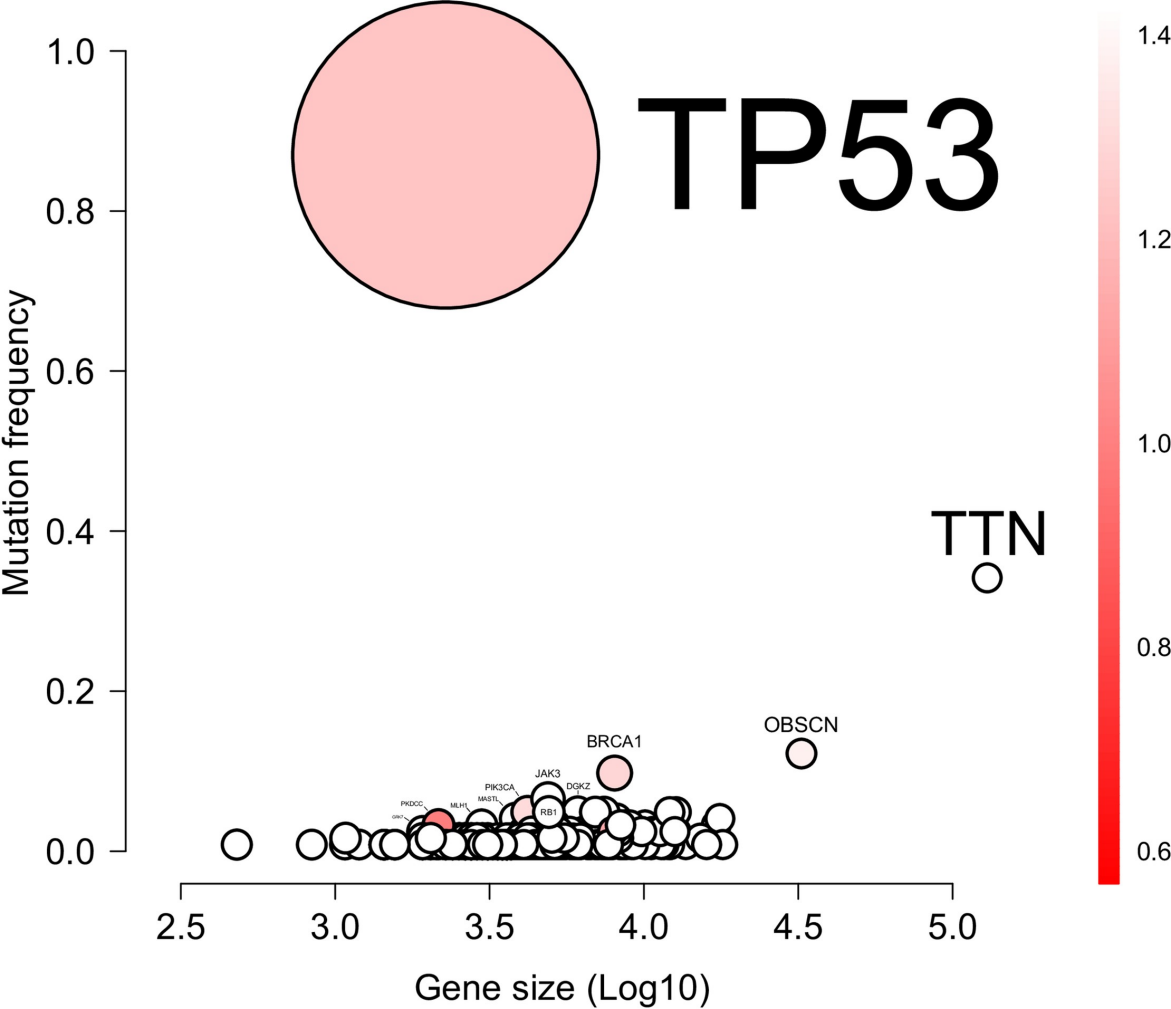
Bubble plot of the mutations found in the discovery ovarian dataset. The x-axis reports the Log10 of the size of the gene; the y-axis reports the mutation frequency of a gene in the dataset. The color bar indicates the specificity of the location of the mutation: the redder the color the more mutations are located at identical positions. The number associated to the color bar is the ratio of the possible mutations sites over the total number of mutations. The size of the bubble is the statistical significance: the bigger the circle the higher significance of the gene.

The next most frequent mutations in the selected HGSOC cases were in *TTN* (N = 40/124, 32%), *OBSC* (N = 15/124, 12%) and *BRCA1* (N = 12/124, 9.7%). Mutations in other genes were identified in less than 10 samples. After correcting for gene size, only *TP53* and *BRCA1* remained significant (adjusted p-value<0.05) (Fig 2).

### Kinome sequencing reveals JAK3 as mutated gene in HGSOC

*JAK3* (N = 8/124, 6.4%) was the most frequently mutated kinase gene with a trend toward significance after correcting for gene size (multiple testing q-value = 0.1). All the variants found in *JAK3* were single nucleotide substitutions (details in S4 Table), 5 located in the kinase or pseudo kinase domains (p.L1091P, p.T1022I, p.G888A, p.K733N, p.K550N) and 3 located in the cytokine receptor binding domain (p.P307S, p.P151R, p.R142C). When we assessed the functional impact of these variants using different predictive *in silico* tools (Mutation Assessor [40], PolyPhen2 [41] and SIFT [42]), all three variants located in the kinase domains (p.L1091P, p.T1022I, p.G888A) and the p.R142C variant were predicted to have a damaging/deleterious impact, while the remaining four variants were frequently predicted as benign. All variants were novel (e.g. not previously annotated in public databases) with exception of two variants, both located in the cytokine receptor ligand binding domain (p.P151R, p.R142C), which were known COSMIC mutations (p.P151R-COSM35867, p.R142C-COSM7088133). The COSM35867 mutation was found in hematopoietic and lymphoid tissues and reported as an inherited loss-of-function mutation for myeloproliferative disorder [43,44] and sporadic hemangioblastomas [45]. COSM7088133 was reported by the TCGA as somatic mutation found in bladder urothelial carcinoma with high probability to be pathogenic (FATHMN (http://fathmm.biocompute.org.uk/) score = 0.9). Out of the six variants, one (p.L1091P) was located at the same position as the COSM1211223, a missense mutation predicted to have a deleterious effect in colon cancer [46]. Matched normal samples were available for 7 of the 8 samples with *JAK3* variants: 5 variants were confirmed to be somatic (p.L1091P, p.T1022I, p.G888A, p.K550N), one variant failed the re-sequencing (p.K733N) and one was confirmed to be germline (p.P151R), as expected.

Therefore, based on the re-sequencing results, the validated somatic mutation frequency of *JAK3* in the discovery HGSOC dataset was equal to 4.0% (N = 5/124).

### mRNA expression levels of *PIM1*, *PIM2*, *PIM3* and *JAK3* genes vary among mutated patient samples

In order to assess whether the mutations found in the *JAK3* gene were functional, i.e. could have an effect on the JAK3 protein activity (and therefore on the JAK-STAT pathway activation status) we measured the mRNA expression levels of *JAK3* and the *bona fide* targets of the JAK-STAT signaling pathway, *PIM1*, *PIM2* and *PIM3*.

We quantified the mRNA expression levels of *JAK3*, *PIM1*, *PIM2* and *PIM3* genes in the clinical samples with *JAK3* mutations (PT-111, p.L1091P; PT-049, p.T1022I; PT-024, p.G888A; PT-020, p.K733N; PT-095, p.K550N; PT-151, p.P307S; PT-130, p.P151R; PT-070, p.R142C) and in four clinical samples without *JAK3* mutations (i.e. control samples, PT-102, PT-101, PT-109, PT-123). The control samples were selected based on the availability of total RNA left for the assessment.

**Fig 3:**
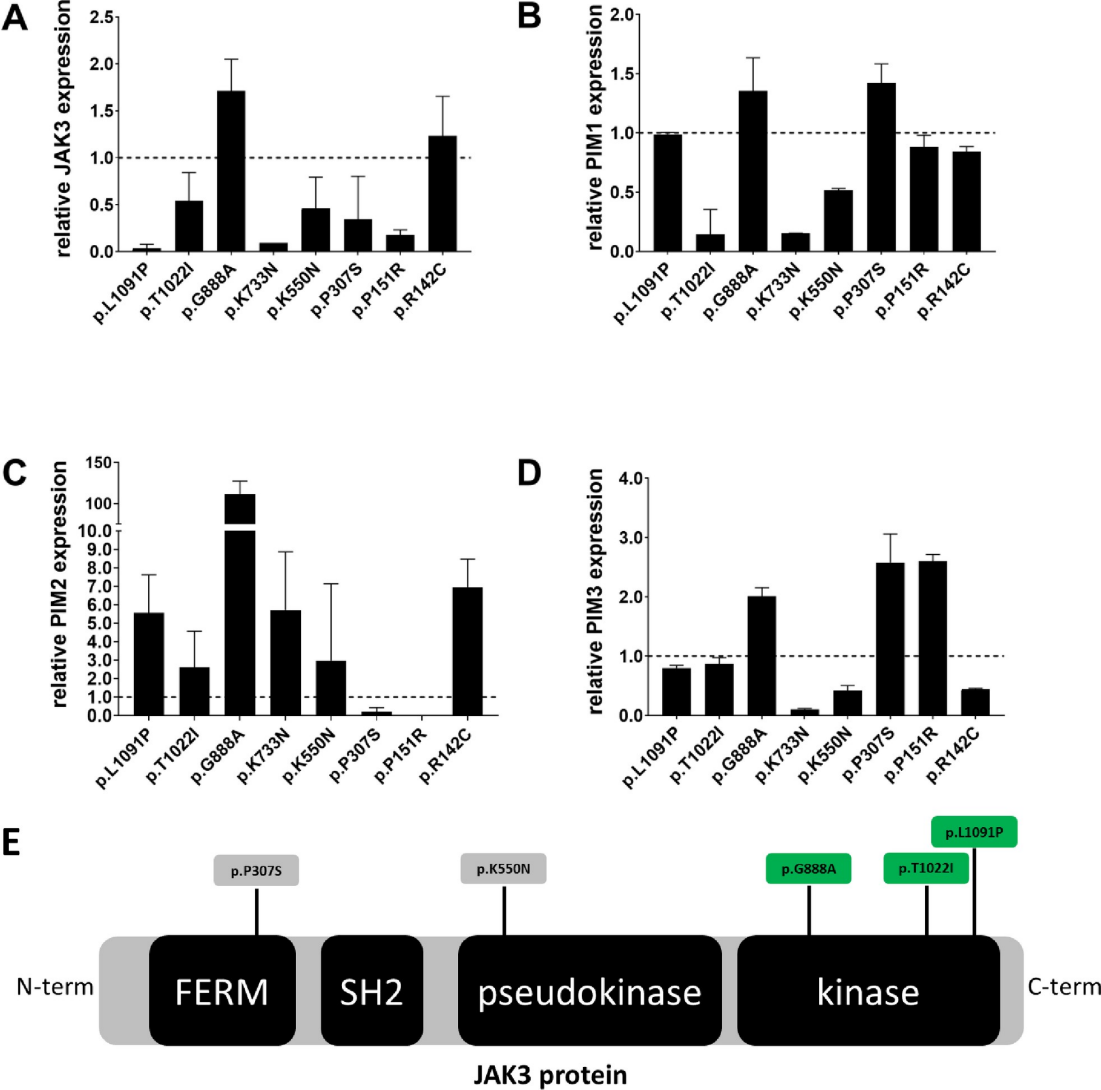
**A-D:** Relative mRNA levels of *JAK3* (A), *PIM1* (B), *PIM2* (C) and *PIM3* (D) in patient tumor samples with *JAK3* mutations (p.L1091P, p.T1022I, p.G888A, p.K733N, p.K550N, p.P307S, p.P151R, p.R142C). Histograms represent the relative abundance of *JAK3* (A), *PIM1* (B), *PIM2* (C) and *PIM3* (D) mRNA in mutated samples compared to their averaged relative mRNA level in four patient tumor samples without *JAK3* mutations (i.e. control samples, PT-102, PT-101, PT-109, PT-123). Dotted black line at y = 1.0, represents the reference control expression level against which the expression fold-change of the mutant samples is calculated. Error bar on the histogram denotes the standard deviation. Tumor samples carry the following mutations: PT_111 = p.L1091P (validated somatic), PT_049 = p.T1022I (validated somatic), PT_024 = p.G888A (validated somatic), PT_020 = p.K733N (validation failed), PT_095 = p.K550N (validated somatic), PT_151 = p.P307S (validated somatic), PT_130 = p.P151R (validated germline), PT_070 = p.R142C (not validated). **E:** Schematic representation of JAK3 protein and its main domains: the four-point-one, Ezrin, Radixin, Moesin (FERM) domain; the Src homology-2 (SH2) domain; and the pseudo kinase and kinase domains. Somatic mutations identified in this study are shown p.G888A, p.T1022I, p.L1091P, p.K550N, p.P307S. Highlighted in green are the mutations functionally assessed in this study. C-term = carboxyl-terminus domain, N-term = amino-terminus domain.

Fig 3A–3D shows the relative mRNA levels of *JAK3*, *PIM1*, *PIM2 and PIM3* (i.e. fold-change expression) compared to averaged relative expression levels of *JAK3*, *PIM1*, *PIM2 and PIM3* genes in the four control samples. There was no statistically significant difference (Mann-Whitney test p-value>0.05) between the expression levels of *JAK3*, *PIM1*, *PIM2* and *PIM3* in the mutated compared to control samples. Nevertheless, the p.G888A (sample PT-024), was associated with higher expression levels of *JAK3*, *PIM1*, P*IM2* and *PIM3*, indicating possible activation of the JAK-STAT signaling pathway.

### *JAK3* p.T1022I mutant identified in HGSOC transform Ba/F3 cells to cytokine-independent growth

To assess whether these mutations could constitutively activate the JAK-STAT pathway *in vitro* we use the Ba/F3 cell line, a murine pro B cell line dependent on Interleukin 3 (IL-3) for proliferation. Ba/F3 cell lines were transduced by retroviral vectors encoding either *JAK3* wild-type or *JAK3* mutants that co-express GFP (see Methods). We focused our analysis on the 3 kinase domain mutations (Fig 3E, highlighted in green) because of their potential clinical relevance as a drug target.

**Fig 4:**
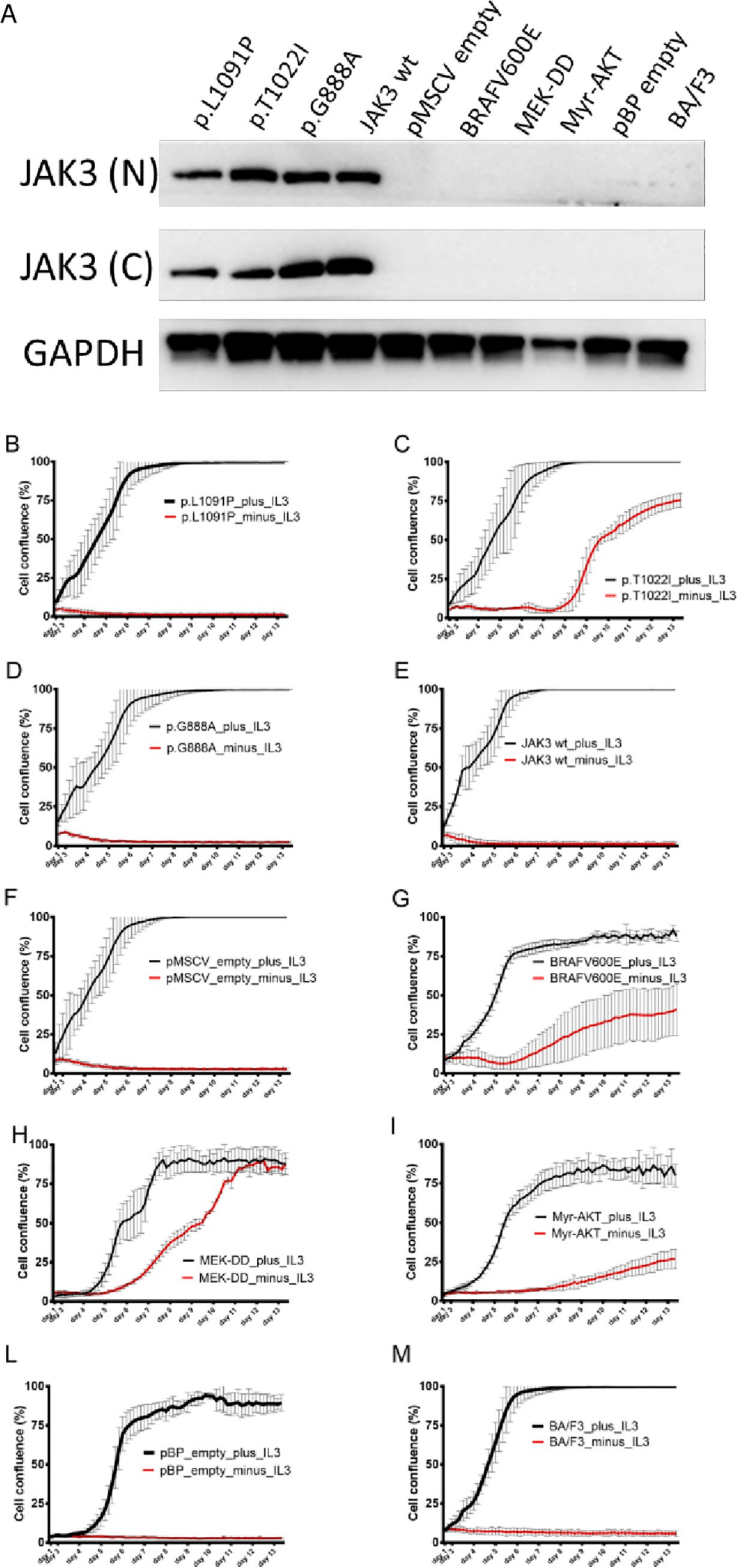
**A:** JAK3 expression in Ba/F3 JA K3 mutant cell lines transduced with JAK3 or control mutant vectors after selection in vitro with IL-3. JAK3 expression was assessed by western blot in Ba/F3 cell lines (selected in vitro with IL-3) with specific anti-JAK3 (N-term and C-term) antibodies and anti-GADPH antibody. **B-M:** Cell proliferation of mutant and wild-type cell lines upon IL-3 withdrawal assessed using the IncuCyte® ZOOM System. Cells were resuspended in 10% FCS RPMI medium with (plus_IL3) and without IL3 (minus_IL3) in a 96-well plate format and followed for 13 days. The x-axis reports time in days, the y-axis reports cell confluence in percentage (%). JAK3(N) = N-terminal antibody; JAK3(C) = C-terminal antibody; GAPDH = glyceraldehyde-3-phosphate dehydrogenase antibody; p.L1091P = JAK3 mutant #1; p.T1022I = JAK3 mutant #2; p.G888A = JAK3 mutant #3; JAK3 wt = JAK3 wild-type; pMSCV empty = pMSCV empty vector; BRAFV600E = pBabe-Puro-BRAF-V600E mutant; MEK-DD = pBabe-Puro-MEK-DD mutant; Myr-AKT = pBabe-Puro-Myr-AKT mutant; pBP empty = pBabe-Puro empty vector; BA/F3 = wild-type Ba/F3 cell lines; IL-3 = Interleukin 3.

To confirm that the transduced Ba/F3 cell lines overexpressed JAK3 at the protein level, we performed western blotting with anti-JAK3 antibodies against the N-terminal and the C-terminal domains of *JAK3* (Fig 4A). This showed that all three *JAK3* mutants and the wild-type *JAK3* Ba/F3 cell line overexpressed JAK3 protein. As expected, Ba/F3 cell lines transduced with non-JAK3 retroviral mutant vectors (such as BRAFV600E, MEK-DD, Myr-AKT) or Ba/F3 wild-type cell line (not-transduced) did not overexpress JAK3 protein.

Upon IL-3 withdrawal, pT1022I mutant cells had IL-3–independent growth of Ba/F3 cells after 13 days of incubation (Fig 4B–4M). In contrast, p.L1091P and p.G888A mutants were not able to induce autonomous cell growth in the absence of IL-3.

*Bona-fide* mutants (BRAFV600E, MEK-DD, Myr-AKT) could confer IL-3 independent proliferation of Ba/F3 cells with Myr-AKT mutant displaying the weakest effect. As expected, *JAK3* wild-type and the empty vector–transduced cells were not able to induce IL-3 independent proliferation.

### JAK3 p.T1022I mutant activates STAT5 and ERK

**Fig 5:**
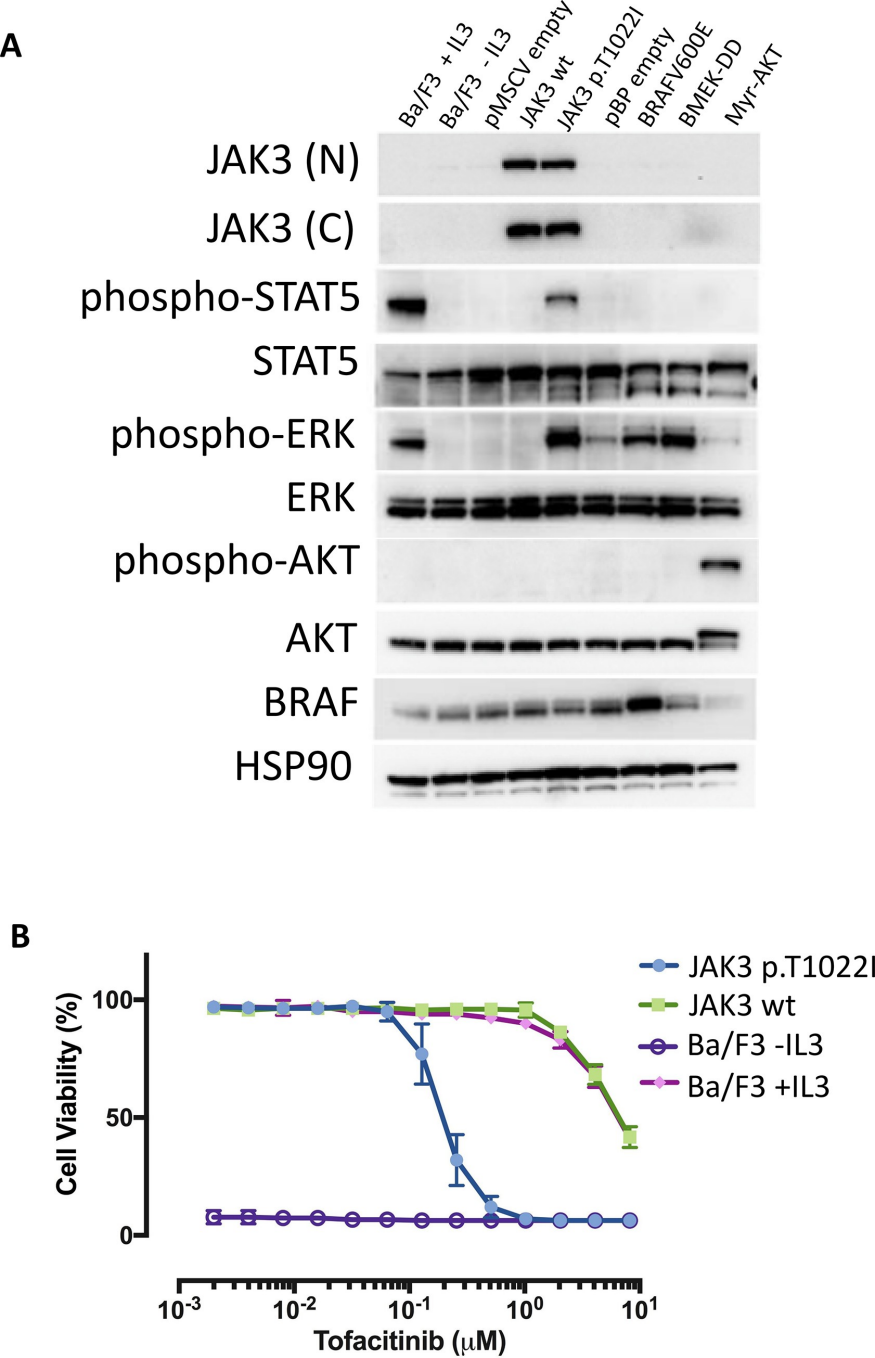
**A**: JAK3 p.T1022I signals through STAT5, and ERK in a cytokine independent manner. JAK3 was able to phosphorylate downstream signaling components STAT5 and ERK. Proteins and phospho-proteins were assessed by western blot in mutants and control Ba/F3 cell lines (after selection in vitro without IL-3) with specific anti-JAK3 (N-term and C-term), anti-phospho STAT5, anti-STAT5, (Tyr694, 9351S), anti-phospho ERK, anti-ERK, anti-phospho AKT, anti-AKT, anti-BRAF and anti-HSP90 antibodies. **B:** 72-hour dose-response assay of JAK3 p.T1022I, JAK3 wild-type and parental (+/- IL3) Ba/F3 cell lines treated with increasing concentration of Tofacitinib. The x-axis reports the drug concentration (μM) and the y-axis the cell viability measured as percentage.

Next, we sought to identify the downstream signaling pathways activated by JAK3 pT1022I in the IL-3 independent Ba/F3 cell lines. A strong phosphorylation of STAT5 and ERK was detected in the JAK3 pT1022I cells, indicating activation of the JAK/STAT pathway *via* STAT5 protein. As expected positive control cells showed phosphorylation of ERK protein (BRAFV600E, MEK-DD mutants) and AKT protein (Myr-AKT mutant) (Fig 5A).

### JAK3 p.T1022I mutant–transformed cells are sensitive to JAK kinase inhibitor Tofacitinib

To assess whether transformation with the p.T1022I mutation could make IL3 independent Ba/F3 cells more sensitive to inhibition of the JAK/STAT pathway than parental Ba/F3 cells, JAK3 pT1022I, JAK3 wild-type and parental Ba/F3 cell lines were treated with increasing concentration of the JAK1/JAK3 inhibitor Tofacitinib and cell viability was measured. IC50 for JAK3 pT1022I was equal to 0.2 μM, while IC50s for the wild-type JAK3 and parental Ba/F3 cells (in the presence of IL3) were both equal to 6.7 μM, indicating that JAK3 pT1022I was approximately 33 times more sensitive to Tofacitinib than controls (Fig 5B). JAK3 pT1022I cells were significantly more sensitive to Tofacitinib than wild-type JAK3 and parental Ba/F3 cells (in the presence of IL3) (Student's paired t-test p-value = 0.008). As expected, parental Ba/F3 cells cultured in absence of IL3 do not proliferate (Fig 5B).

### TAm-Seq reveals 1% *JAK3* and 7%*CDK12* mutation frequency in 463 HGSOC patient samples

We performed independent mutation analysis of *JAK3* using TAm-Seq to accurately define its mutation frequency and potential clinical utility using a cohort of 463 HGSOC patients. We included the *CDK12* gene, as this was the most significant mutated kinase gene identified by the TCGA in HGSOC at a frequency of 3% [4].

We found 18 variants in the *JAK3* gene of which 17 unique and 1 recurrent in four different patients. The majority were missense/nonsense/frameshift variants (N = 15/18). Out of the 18 variants, five were confirmed to be somatic and 9 to be germline after sequencing the matched normal samples. We were unable to validate three variants owing to lack of matched normal DNA. We could therefore define a *JAK3* mutation frequency of 1% (5/463). The somatic mutations were not clustered in a specific region of the gene. A detailed summary of the *JAK3* variants is reported in S5 Table. It should be noted, that among the 18 variants identified in the *JAK3* gene, none of the 8 variants from the kinome sequencing analysis were found.

**Fig 6:**
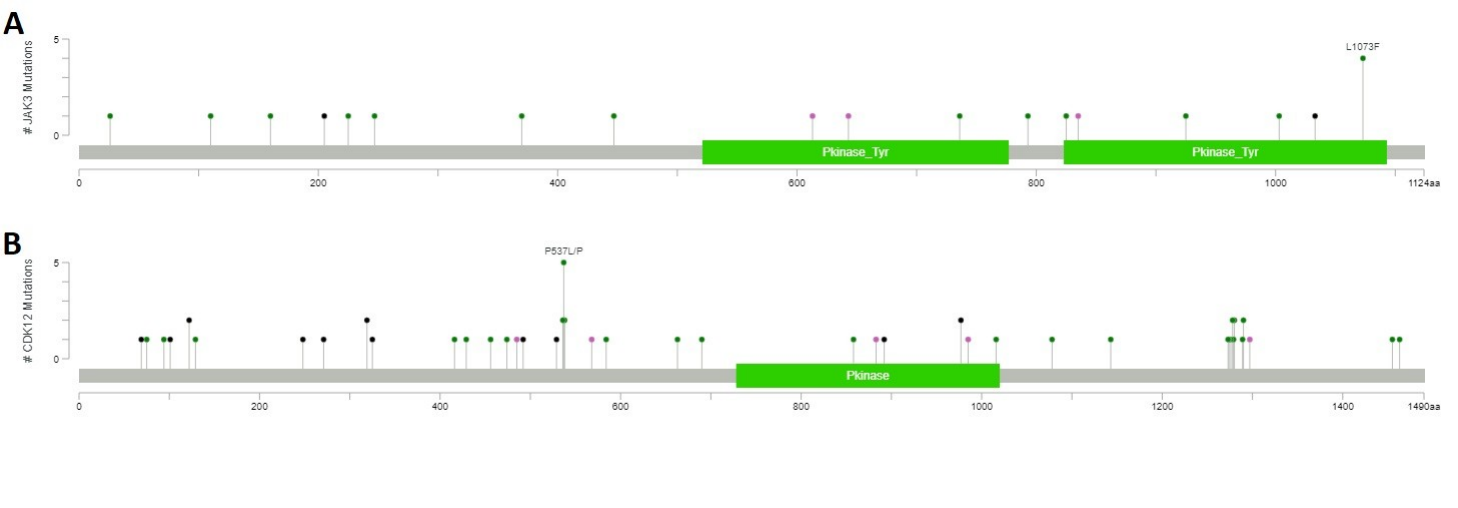
Mutation maps of JAK3 (**A**) and CDK12 (**B**) proteins. Diagram circles (“lollipop plots”) represent the variants found in *JAK3* and *CDK12* genes in the validation series of 463 ovarian samples. The circles are colored with respect to the corresponding mutation types: green = Missense mutations, black = Truncating mutations (Nonsense, Frameshift insertion/deletion), purple = Other types of mutations. The x-axis report the amino acid number, the y-axis reports the frequency of the mutation. Mutation maps were generated using the MutationMapper tool from the cBioPortal database.

We found 47 variants in the *CDK12* gene, of which 25% (N = 12/47) were loss-of-function mutations (N = 4 frameshift, N = 5 deletion, N = 3 nonsense). There were 7 recurrent mutations, of which two were nonsense mutations (one in the arginine/serine-rich domain at Y319* and one in the kinase domain Q977*), five missense mutations, (P536S, P537L, P538S, P1280S) and one silent mutation (P537P) were all located in the proline-rich domain. After sequencing the matched normal samples, 33 of 47 variants were confirmed somatic (70%). We were unable to validate eight variants owing to lack of matched normal DNA. The somatic mutations were not clustered in a specific region of the gene. We could therefore define a *CDK12* mutation frequency of 7% (33/463). A detailed summary of the *CDK12* variants is reported in S5 Table. Fig 6 shows the mutation maps of *JAK3* and *CDK12* genes.

## Discussion

In this study we carried out an in-depth kinome mutation analysis of 127 serous ovarian carcinomas, of which 124 were confirmed to be high-grade serous carcinoma (HGSOC), using a DNA kinome-targeted enrichment strategy followed by high-throughput sequencing. In addition to the kinase genes, we also screened 48 *bona-fide* cancer-related genes, which could play a role in the pathogenesis of ovarian cancer as well. Using this enrichment strategy, we obtained 96% of the target genes with at least 30× coverage, of which 50% had at least 150× coverage and therefore we could detect DNA variants present also at low allele frequency.

In 2011, a key study of The Cancer Genome Atlas (TCGA) research network shed light on the genetic alterations driving HGSOC. The researchers employed a variety of high throughput technologies to systematically catalogue molecular aberrations in about 500 HGSOC cases. Mutation analysis in the TCGA study revealed that HGSOC had a simple mutational profile confirming ubiquitous mutation of *TP53* with other genes mutated at low frequency. More recently, Patch and colleagues confirmed that somatic point mutations in driver genes are infrequent in primary HGSOC with *TP53* being the prevalent (and only significantly) mutated gene [7]. However, the average sequencing depth of these studies was lower (maximum 30–50×) than what we achieved using our targeted approach and this could have limited the discovery of mutations with low allele frequency (smaller than 5%). We therefore initiated this study to validate and to extend what was known on the mutational profile of this type of epithelial ovarian cancer. Consistent with previous reports, in our patient set the most frequently mutated gene was *TP53* with 97% (N = 120/124) HGSOC samples mutated in our study.

Among the top 15 genes mutated in our sample set, the majority were kinase genes (N = 11/15, 73%) reflecting strong enrichment in our sequencing panel (Fig 1). Some genes were previously reported by the TCGA as significantly mutated in HGSOC, such as *BRCA1*, *BRCA2*, *RB1* and *CDK12*. Other genes significantly mutated in the TCGA HGSOC cohort, were either not included in our target gene panel (*CSMD3*, *FAT3*, *GABRA2*) or were mutated at lower frequency in our dataset (1.6% *NF1*). Interestingly, the average mutation frequency of the kinase genes mutated in our panel was 1.5% (N = 1.9/124), which was slightly lower than the mutation frequency observed for the non-kinase genes (1.6%, N = 2.0/124) after excluding the highest mutated expected genes, *TP53* and *BRCA1*. This result indicates that kinase genes are indeed mutated at low frequency in HGSOC, compared to commonly mutated genes such as *BRCA1* (N = 12/124, 9.7%), *BRCA2* (N = 6/124, 4.8%) or *RB1* (N = 6/124, 4.8%). Taken together, our results independently validate previous findings of the TCGA highlighting that HGSOC has a very restricted number of recurrent mutations with *TP53* as the main oncogenic driver.

Unexpectedly, we identified the *JAK3* gene as the most frequently mutated gene after *TP53*, *TTN*, *OBSC* and *BRCA1* with a trend toward significance after correcting for multiple testing (adjusted p-value = 0.1). Out of the eight *JAK3* mutations, only one validated as germline. This is in line with a previous study from O’Shea and colleague where this mutation was detected as inherited in severe combined immune deficiency (SCID) patients [44]. However, other studies [43,45] report this mutation as somatic suggesting that loss-of function mutations of *JAK3* can be also somatically acquired in other diseases (sporadic hemangioblastomas, Down syndrome, acute megakaryoblastic leukemia). Out of the three mutations found in the *JAK3* kinase domain, the p.T1022I mutation transformed Ba/F3 cells to cytokine-independent growth through activation of the JAK/STAT pathway *via* STAT5 protein. Importantly, p.T1022I Ba/F3 transformed cells were highly sensitive to Tofacitinib, a JAK1/3-selective inhibitor when compared to non-transformed cells. Of note, based on *in silico* prediction tools, all three kinase mutations were likely to be deleterious, however only the p.T1022I resulted in oncogenic transformation of our *in vitro* model. This highlights the importance of functional assays to further select candidate somatic mutations coming from high throughput mutation screening.

We could hypothesize that ovarian tumor cells acquiring this mutation would become addicted to JAK-STAT activated signaling pathways for survival and therefore sensitive to JAK-STAT pathway inhibition. Therefore, based on our findings, the p.T1022I mutation could be considered as potential biomarker for treatment sensitivity. Nevertheless, we did not detect the p.T1022I mutation in the 463 HGSOC validation cohort, indicating that this mutation is most probably a very rare event in HGSOC. Also, JAK3 T1022I mutations are not present, after review in the cBioPortal database (https://www.cbioportal.org/) which contains over 178 manually curated studies, including the TCGA datasets. Taken together, we conclude that JAK3 T1022I mutations have very low occurrence, but based on our functional data should be considered as potentially actionable if detected.

The TCGA study identified *CDK12* as the most significantly mutated kinase in HGSOC. The *CDK12* gene encodes the cyclin dependent kinase 12 that displays CTD (polymerase II carboxy-terminal domain) kinase activity and is required for RNA splicing [47,48]. In light of its mutation pattern, *CDK12* can be classified as tumor suppressor-like gene. Previous functional studies have confirmed that *CDK12* kinase domain mutations inhibit its catalytic activity and cells with catalytically inactive CDK12 have impaired homologous recombination (HR) [49,50]. Missense as well as stop-gain and indel mutations in *CDK12* can induce allosteric structural defects that alter formation and activity of the Cdk12/CycK complex [51]. Inactivation of this complex in ovarian cancer cells disrupts HR and increased sensitivity to DNA crosslinking agents and poly ADP-ribose polymerase (PARP) inhibitors [49,50]. *CDK12* inactivating mutations are casually linked to large tandem duplications and genomic instability [9,52].

Using capture sequencing, we found *CDK12* mutations in 5/124 tumor samples (4% mutation frequency) which is comparable to that originally reported by the TCGA in 2011 (9/316, 3%) and recently reported by Berger and colleagues (21/523, 4%) [53]. Using high sensitivity tagged amplicon sequencing (Tam-Seq) [30,31] we showed that *CDK12* gene was somatically mutated in 7% (33/463) of the validation cohort. Mutations were not clustered in a specific region of the gene and approximately one third (12/33, 36%,) were loss-of-function (Fig 6B). This mutation pattern is consistent with the putative role tumor suppressor of *CDK12*, where loss of function mutations are scattered throughout the protein coding sequence. To our knowledge, this is the first study that performed an in-depth screening of the *CDK12* gene in a large patient dataset. Considering the comparatively high frequency of *CDK12* mutation in our study (7%), *CDK12* should be added in the list of somatic alterations routinely assayed in women with HGSOC as loss of function mutations predict altered homologous recombination and potential response to PARP inhibitor therapy. However, further functional and clinical characterization is required to define which non-synonymous mutations may also characterize HR deficient tumors [51,52].

In summary, our study highlights the importance of high-depth sequencing analyses and functional characterization to identify low-frequency mutated alleles that could have predictive value in the clinic as biomarkers for patient treatment selection.

## Supporting information

S1 File Detailed description of the *TP53* mutation screening performed in the study. (DOCX)

S1 Fig **A.** Total read count for 12 pooled captured libraries (representative example) sequencing using the kinome panel on the HiSeq 2000 instrument: the x-axis reports the sample names and the y-axis the total number of reads (million). **B.** Percentage over the total read number of reads on target for 12 pooled libraries: the x-axis reports the sample names and the y-axis the percentage of reads on target. **C.** Coverage assessment for 12 pooled libraries: the x-axis reports the mean coverage and the y-axis the percentage of regions on target covered **D.** Proportion of bases on target x-fold coverage, average of 20 samples.
(TIF)

S2 Fig Number of variant per Megabase (Mb) in the discovery ovarian set (N = 127). The x-axis reports the number of samples; the y-axis reports the number of the somatic variants (black) and the germline variants (orange) per Mb.
(TIF)

S3 Fig Mutation rate of discovery ovarian set (N = 127). The x-axis reports the fraction of candidate somatic mutated genes (rate) over the kinome gene set (N = 597). The y-axis reports the density of the samples per gene mutation rate.
(TIF)

S4 Fig Mutation maps of TP53 protein. Diagram circles (“lollipop plots”) represent the variants found in *TP53* gene. The circles are colored with respect to the corresponding mutation types: green = Missense mutations, black = Truncating mutations (Nonsense, Frameshift insertion/deletion), purple = Other types of mutations. The x-axis report the amino acid number, the y-axis reports the frequency of the mutation. Mutation maps were generated using the MutationMapper tool from the cBioPortal database.
(TIF)

S5 Fig Flow diagram of the samples included in the study. From a total of 153 HGSOC samples, 26 were excluded because of low tumor sample cellularity and 127 samples left for the kinome sequencing analysis. After a secondary pathology revision, three samples were excluded because of different histology than high grade serous.
(TIF)

S1 Table Clinical characteristics of the discovery ovarian set used for the kinome mutation analysis (n = 127). (DOCX)

S2 Table **A**: Genes contained in the SureSelect Human Kinome 3.2Mb bait set, **B**: List of 798 candidate somatic variants found in the discovery ovarian set with the kinome mutation analysis.
(XLSX)

S3 Table TP53 mutation found in the discovery ovarian set (N = 127). For each patient sample is reported if TP53 mutation is present (YES/NO) and which TP53 mutation was found, *Revised with TAm Seq.
(XLSX)

S4 Table List of JAK3 variants found in the discovery ovarian set (N = 127). (XLSX)

S5 Table **A:** *JAK3* variants found in the validations ovarian set (N = 463), **B**: *CDK12* variants found in the validations ovarian set (N = 463).
(XLSX)

S1 Raw images Original uncropped blot images for the blots reported in Fig 4A (page 1) and Fig 5A (page 2–6). Images are showed before (called colorimetric) and after exposure (called blot).
(PDF)
