# Supplementary material for: Kinome capture sequencing of high-grade serous ovarian carcinoma reveals novel mutations in the JAK3 gene
Source: PLoS One. 2020 Jul 8;15(7):e0235766. doi: 10.1371/journal.pone.0235766 (PMC7343160; doi:10.1371/journal.pone.0235766)
Supplement: S1 File — (DOCX) [file pone.0235766.s001.docx]

**Supplementary File 1**

By kinome capture sequencing we found 109 samples out of 127 with a somatic mutation in TP53 (85% mutation rate). This percentage was lower than expected from previous studies4,8,32 and therefore we performed an in depth analysis of TP53 as single gene. In order to exclude false-negative cases among the 18 samples with no TP53 mutation, we revised first the unfiltered mutation data and the TP53 genomic sequence using the Integrative Genomics Viewer (IGV) tool27. Out of 18 samples, 8 showed a mutation in TP53, which was previously missed due to our filter strategy. The remaining 10 samples were assessed for TP53 mutation status using TAm-Seq (see Material and methods), which gives extremely high read coverage (1000-5000x) per selected amplicon. Three out of the 10 samples were found TP53 mutated resulting in an overall TP53 mutation rate for the ovarian set to 94% (N=120/127). Table S3 lists the TP53 mutations found. A total of 99 TP53 unique variants were found, of which 71 known COSMIC mutations (7 indel and 65 point mutations), 7 known in the IARC TP53 database (4 indel and 3 point mutations) and the remaining 21 novel variants (20 indels and 1 recurrent point mutation). Of the 71 COSMIC mutations, 12 point mutations were recurrent (2 -5 times). Majority of the mutations (90/99) were located in the P53 DNA-binding domain (Figure S4). Seven samples were confirmed to be negative for TP53 mutation. To further confirm this, we performed a second pathology review of the 7 mutation-negative cases, which resulted in the re-classification of 2 cases as low-grade serous ovarian carcinoma (LGSOC) and one case as mucinous ovarian carcinoma. In support of the second pathology review, we found that these 3 samples (PT_126, PT_131, PT_150), had respectively p.Q61R NRAS, p.G12D NRAS and p.G13D KRAS mutations. The original diagnosis of HGSOC was confirmed in the remaining 4 cases. In summary, the overall TP53 mutation rate for selected HGSOC cases in our set was 96.8% (N=120/124).
