# Supplementary figures and images for: Kinome capture sequencing of high-grade serous ovarian carcinoma reveals novel mutations in the JAK3 gene
Source: PLoS One. 2020 Jul 8;15(7):e0235766. doi: 10.1371/journal.pone.0235766 (PMC7343160; doi:10.1371/journal.pone.0235766)

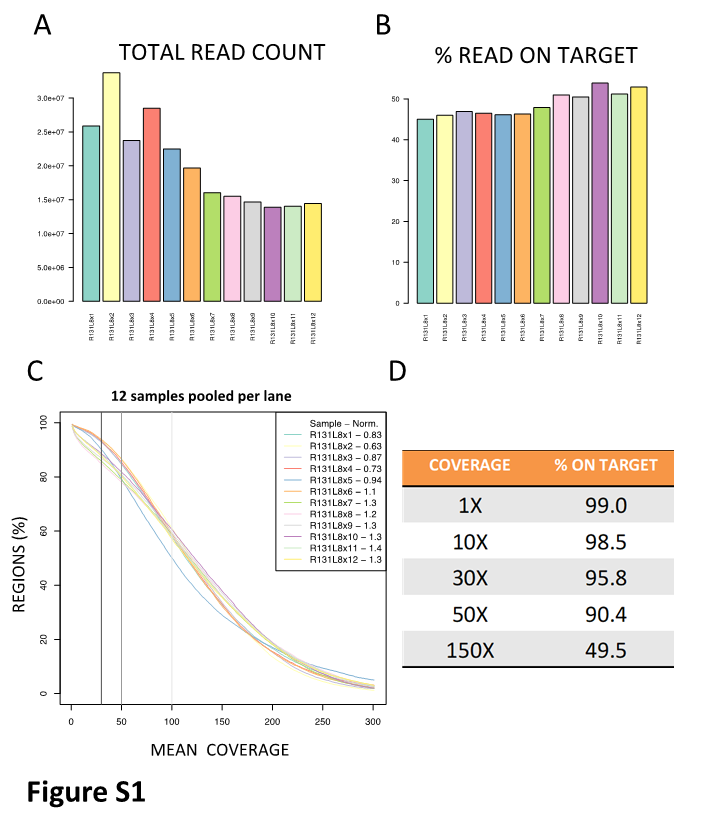

Supplement: S1 Fig — A. Total read count for 12 pooled captured libraries (representative example) sequencing using the kinome panel on the HiSeq 2000 instrument: the x-axis reports the sample names and the y-axis the total number of reads (million). B. Percentage over the total read number of reads on target for 12 pooled libraries: the x-axis reports the sample names and the y-axis the percentage of reads on target. C. Coverage assessment for 12 pooled libraries: the x-axis reports the mean coverage and the y-axis the percentage of regions on target covered D. Proportion of bases on target x-fold coverage, average of 20 samples. (TIF) [file pone.0235766.s002.tif]

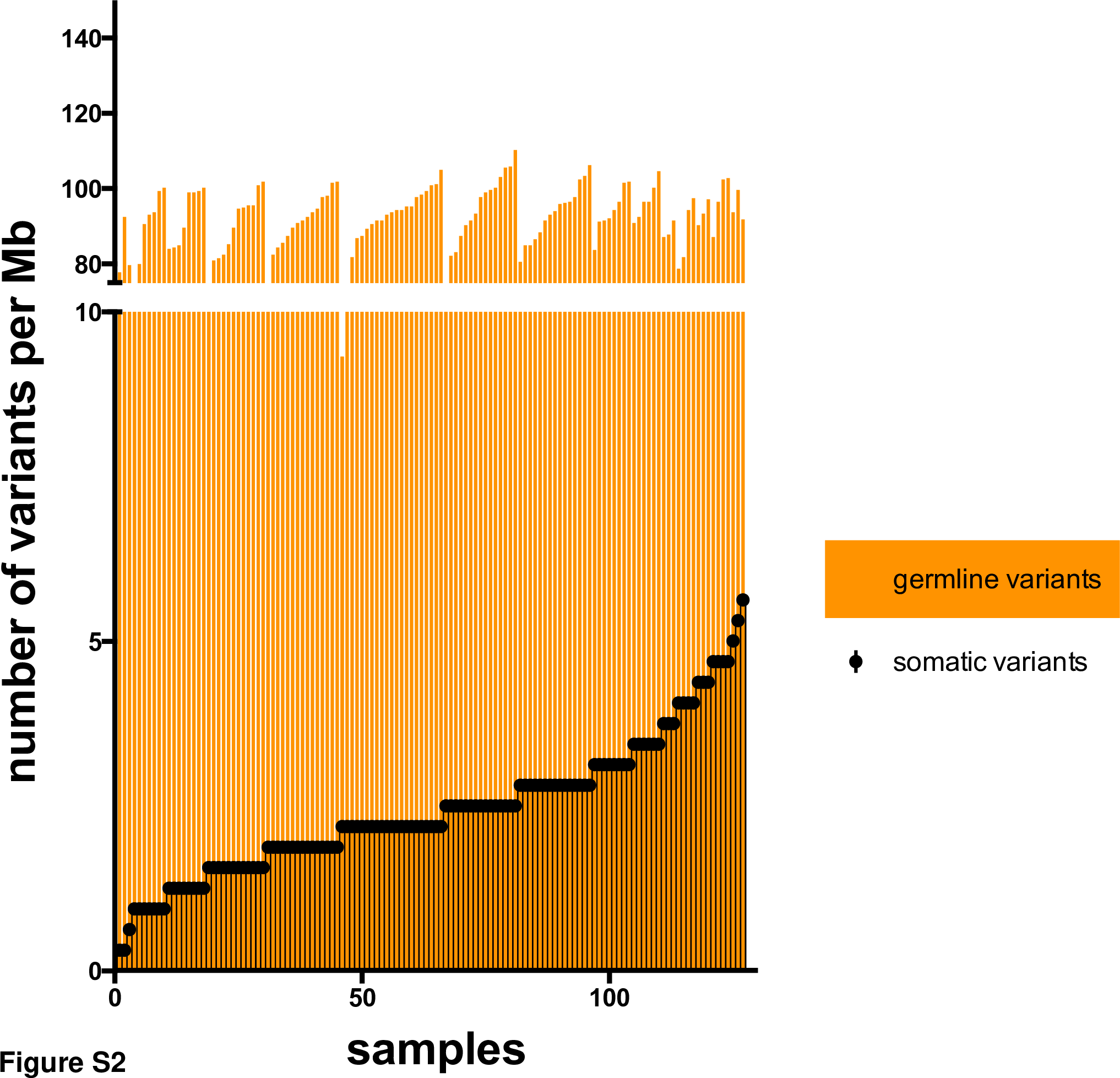

Supplement: S2 Fig — The x-axis reports the number of samples; the y-axis reports the number of the somatic variants (black) and the germline variants (orange) per Mb. (TIF) [file pone.0235766.s003.tif]

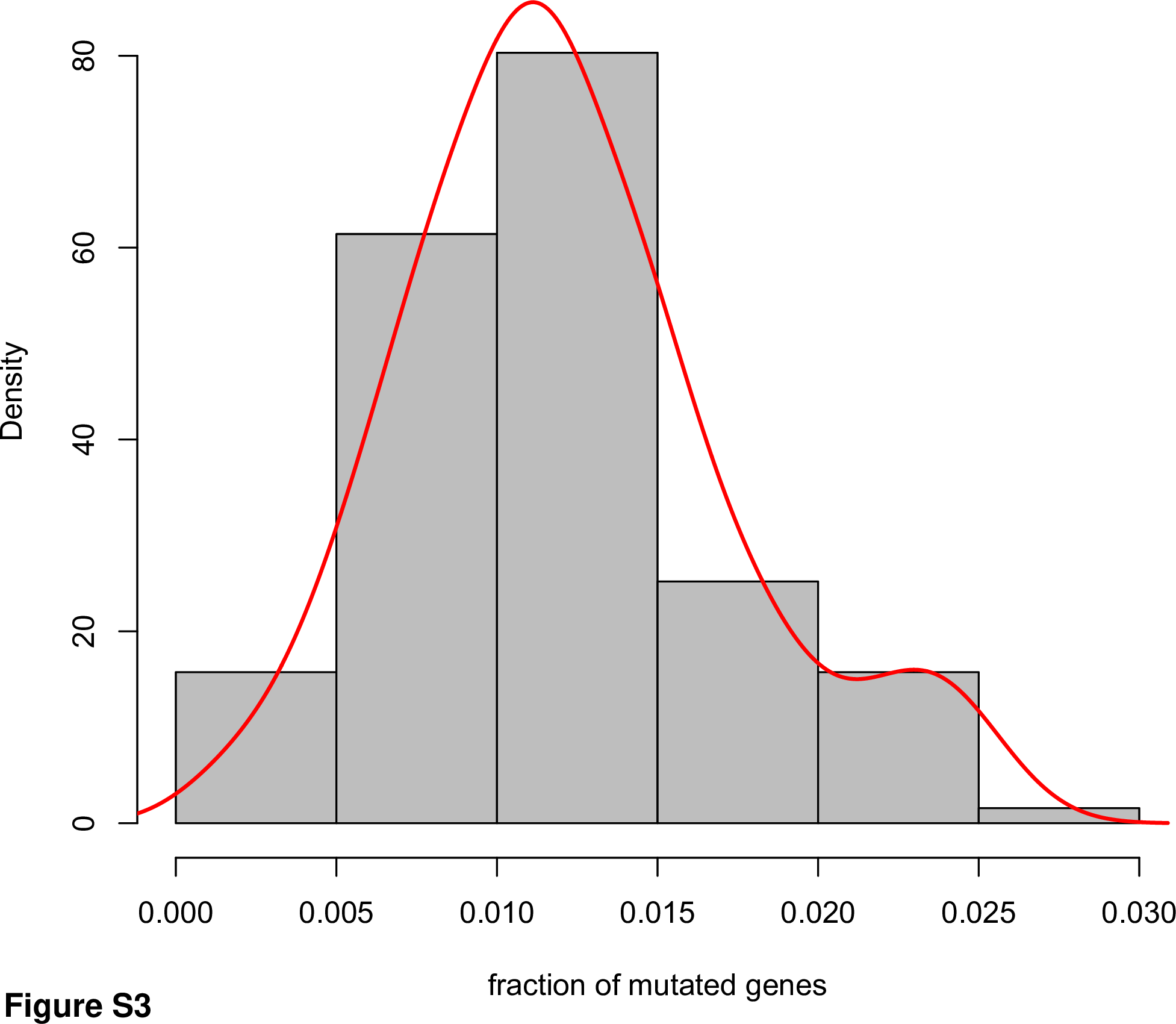

Supplement: S3 Fig — The x-axis reports the fraction of candidate somatic mutated genes (rate) over the kinome gene set (N = 597). The y-axis reports the density of the samples per gene mutation rate. (TIF) [file pone.0235766.s004.tif]

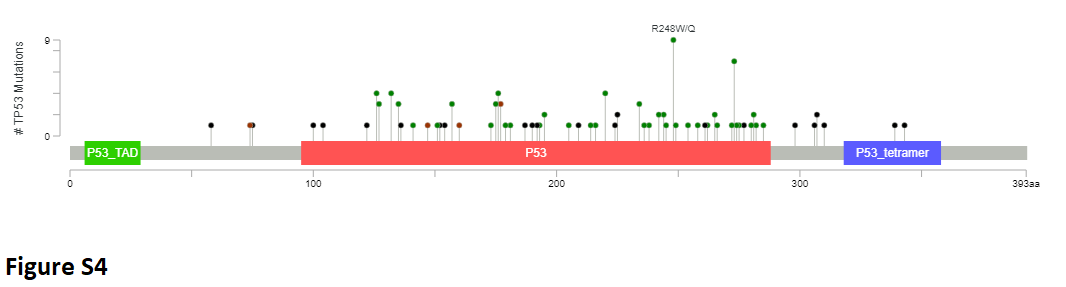

Supplement: S4 Fig — The circles are colored with respect to the corresponding mutation types: green = Missense mutations, black = Truncating mutations (Nonsense, Frameshift insertion/deletion), purple = Other types of mutations. The x-axis report the amino acid number, the y-axis reports the frequency of the mutation. Mutation maps were generated using the MutationMapper tool from the cBioPortal database. (TIF) [file pone.0235766.s005.tif]

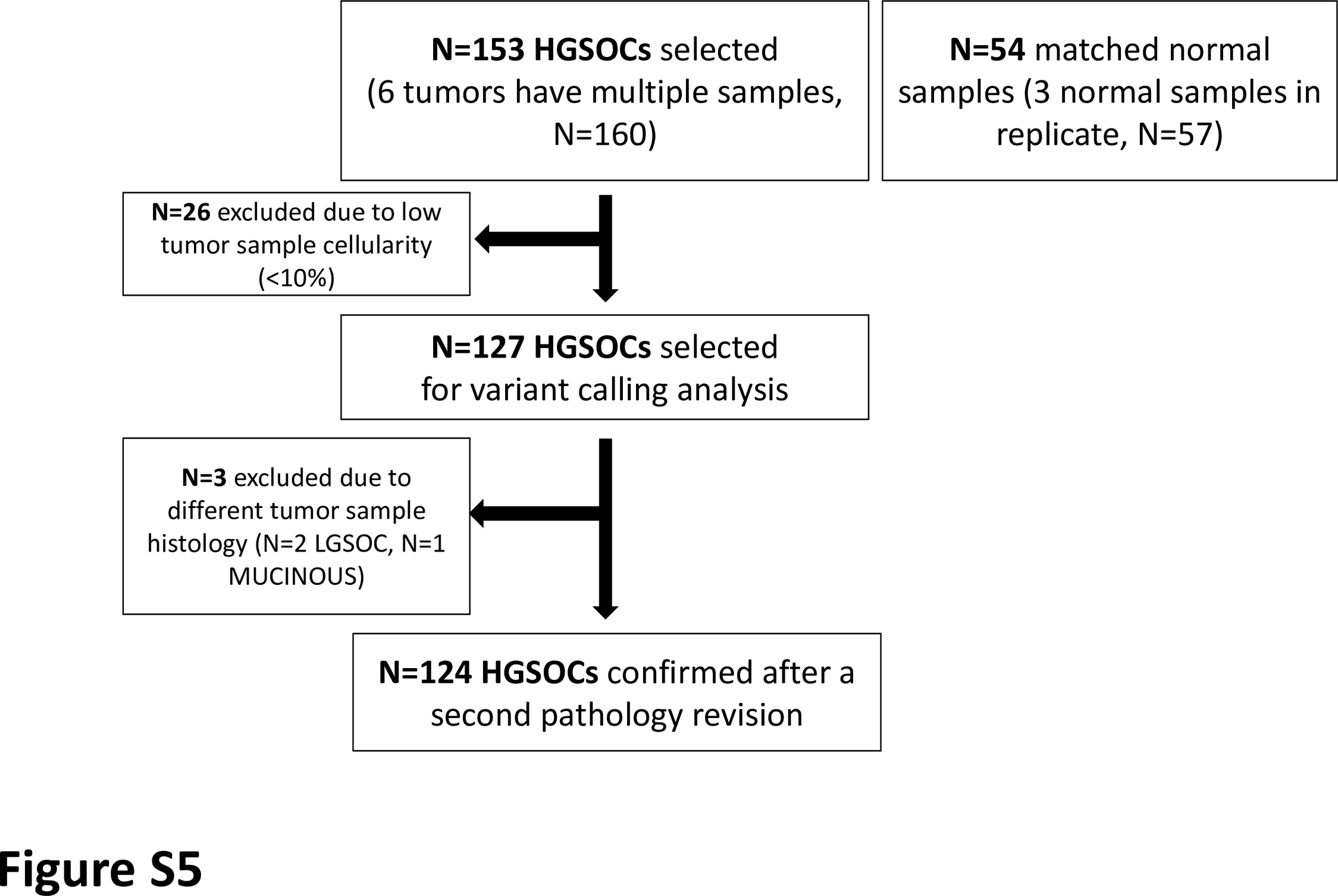

Supplement: S5 Fig — From a total of 153 HGSOC samples, 26 were excluded because of low tumor sample cellularity and 127 samples left for the kinome sequencing analysis. After a secondary pathology revision, three samples were excluded because of different histology than high grade serous. (TIF) [file pone.0235766.s006.tif]
