# Supplementary material for: Kinome capture sequencing of high-grade serous ovarian carcinoma reveals novel mutations in the JAK3 gene
Source: PLoS One. 2020 Jul 8;15(7):e0235766. doi: 10.1371/journal.pone.0235766 (PMC7343160; doi:10.1371/journal.pone.0235766)
Supplement: S1 Table — (DOCX) [file pone.0235766.s007.docx]

| Variable | N |
| --- | --- |
| Total | 127 |
| Tumor collection |  |
| Surgery/diagnosis | 90 |
| IDS | 32 |
| Relapse | 5 |
| PFS (months) |  |
| Median | 14 |
| Range | 0-132 |
| OS (months) |  |
| Median | 30 |
| Range | 1-213 |
| Age (years) |  |
| Median | 62 |
| Range | 37-84 |
| Stage |  |
| III | 87 |
| IV | 30 |
| Other | 10 |
| Residual disease* |  |
| No residual disease | 20 |
| ≤ 1cm | 41 |
| >1cm | 61 |
| Other | 5 |

**Table S1**: Clinical characteristics of the discovery ovarian set used for the kinome mutation analysis (n=127)

IDS=Interval Debulking Surgery, PFS=Progression Free Survival, OS=Overall Survival

*Residual of the tumor after primary surgery
