# Supplementary material for: Kinome capture sequencing of high-grade serous ovarian carcinoma reveals novel mutations in the JAK3 gene
Source: PLoS One. 2020 Jul 8;15(7):e0235766. doi: 10.1371/journal.pone.0235766 (PMC7343160; doi:10.1371/journal.pone.0235766)

Figure 4- blot

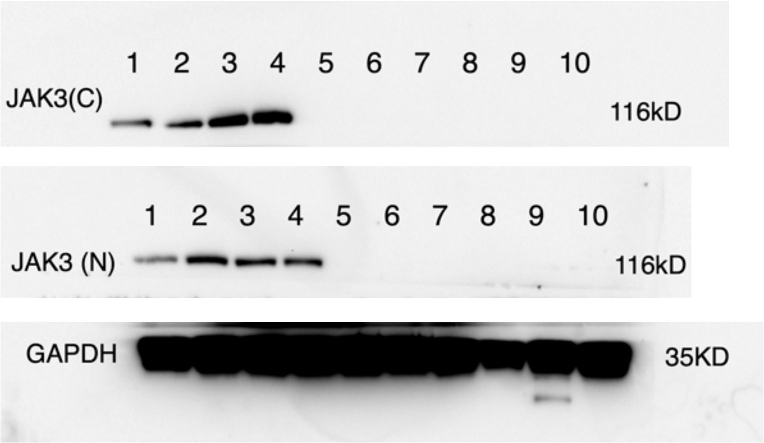

Figure 4- colorimetric

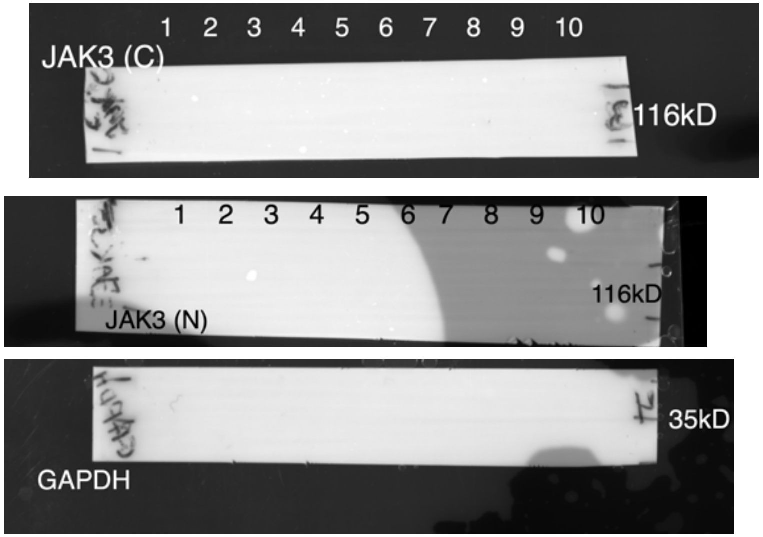

Figure 4- blot and colorimetric merged

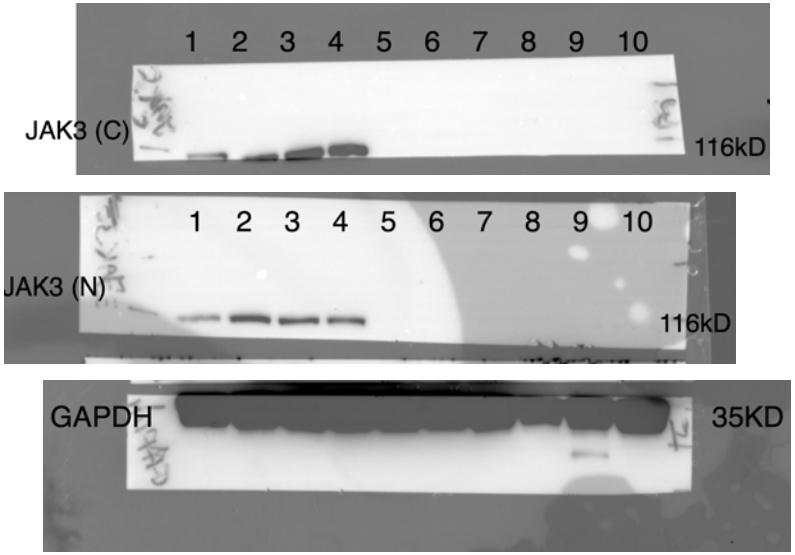

Figure 5- part 1, blot (high and low exposure)

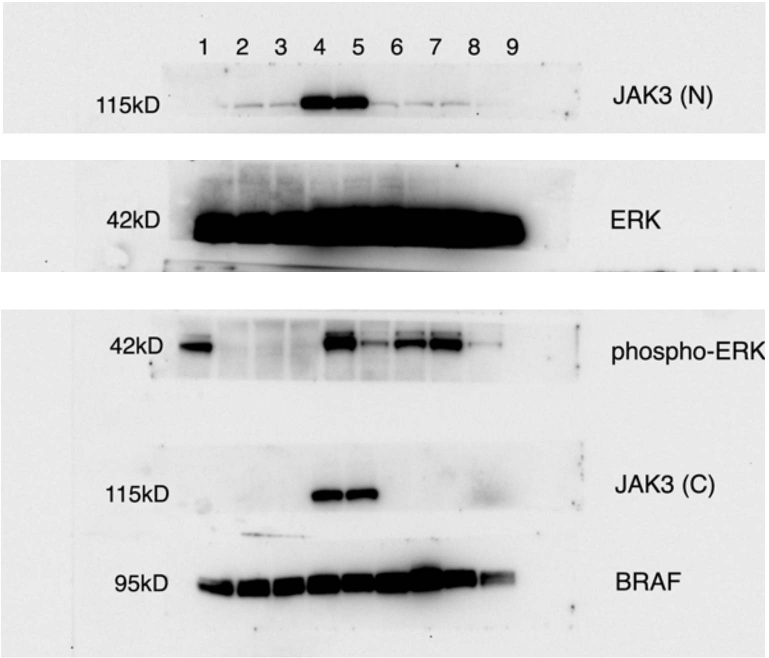

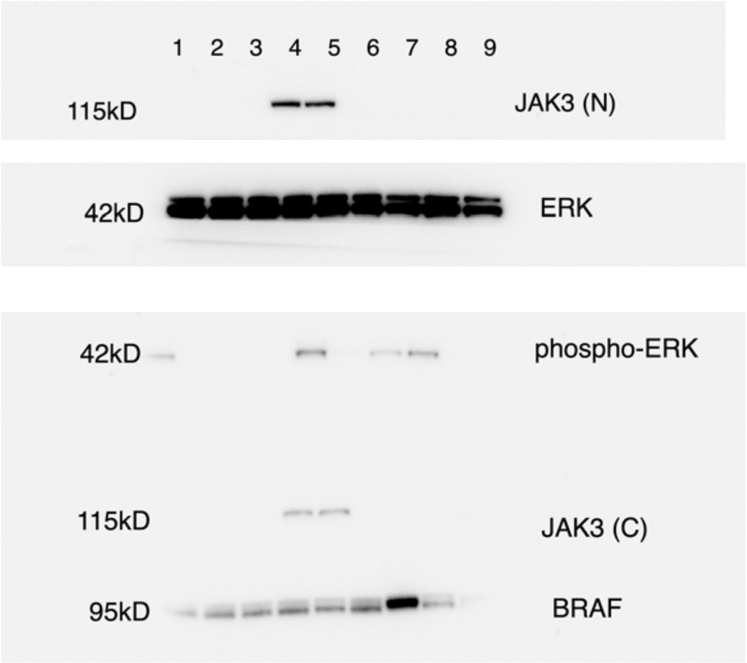

Figure 5- part 1, colorimetric

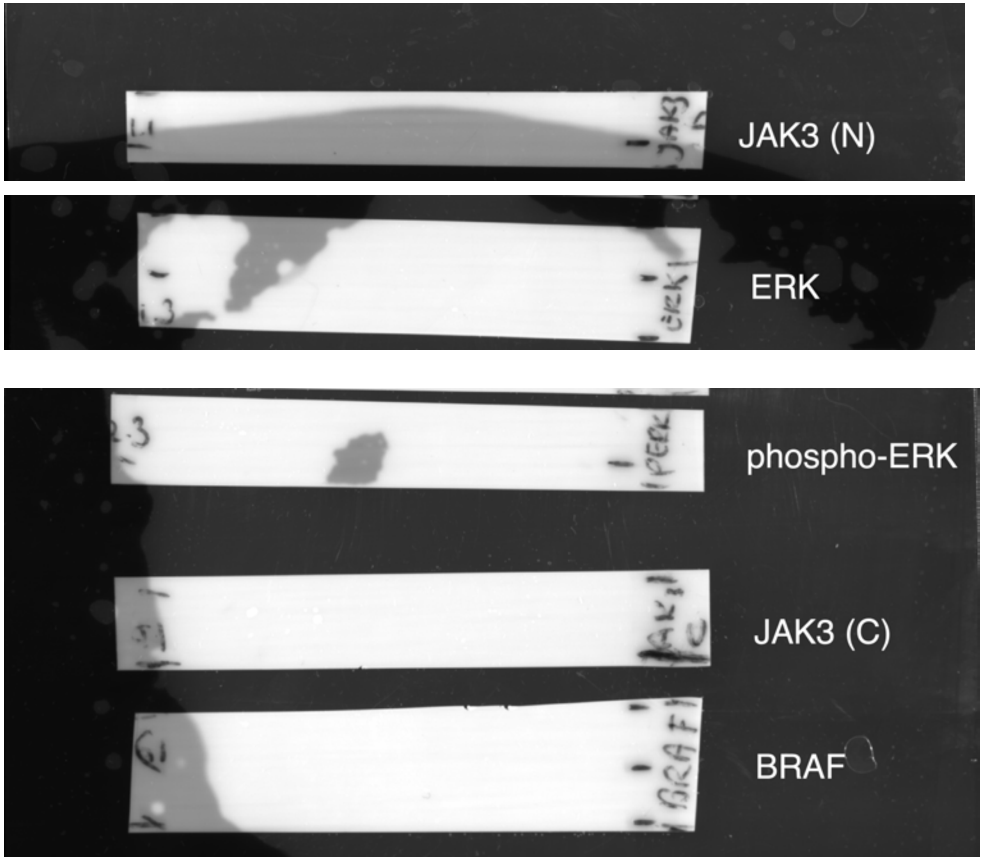

Figure 5- part 1, blot and colorimetric merged (high and low exposure)

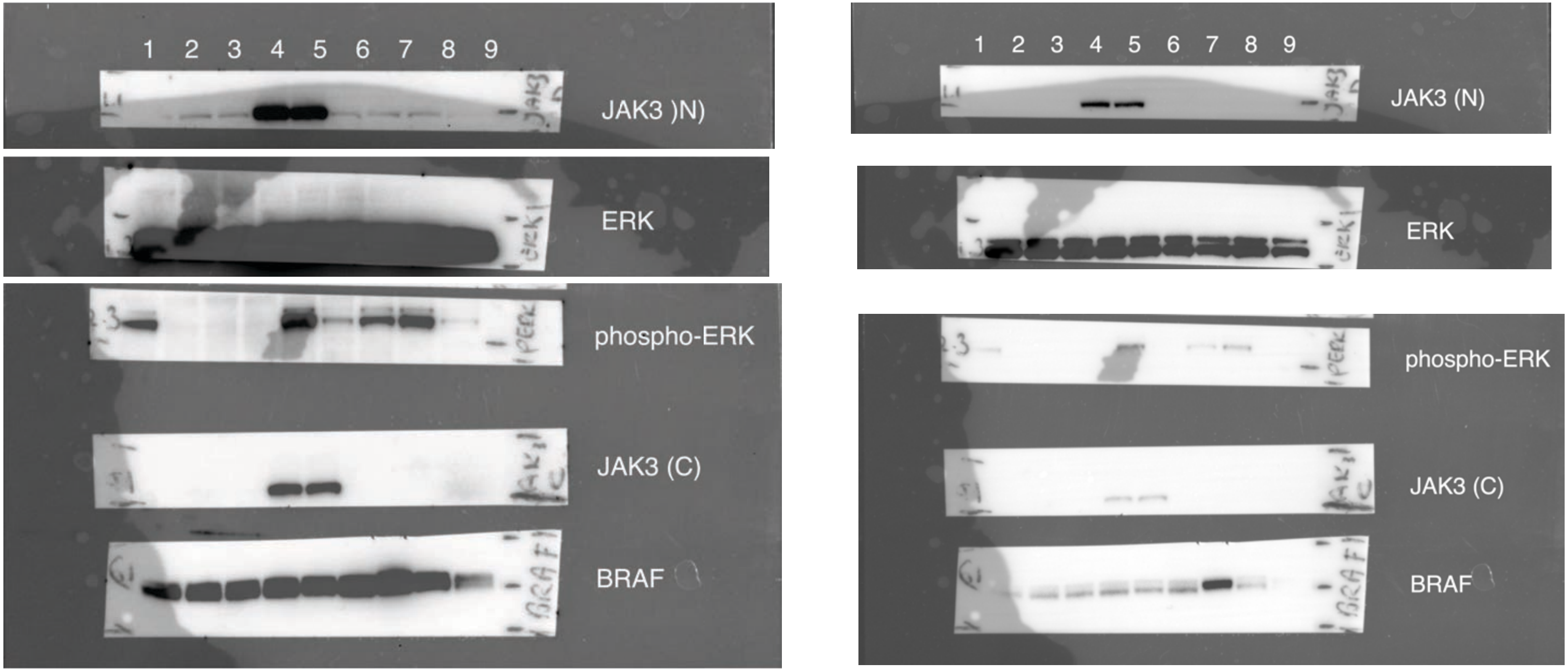

Figure 5- part 2, colorimetric

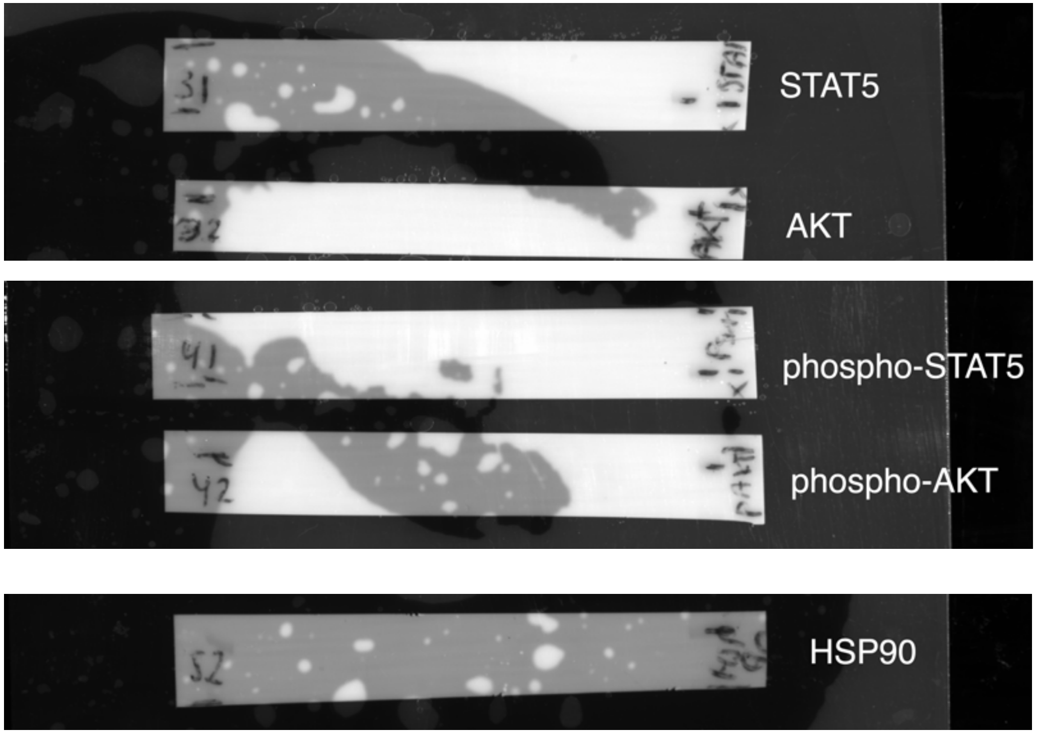

Figure 5- part 2, blot

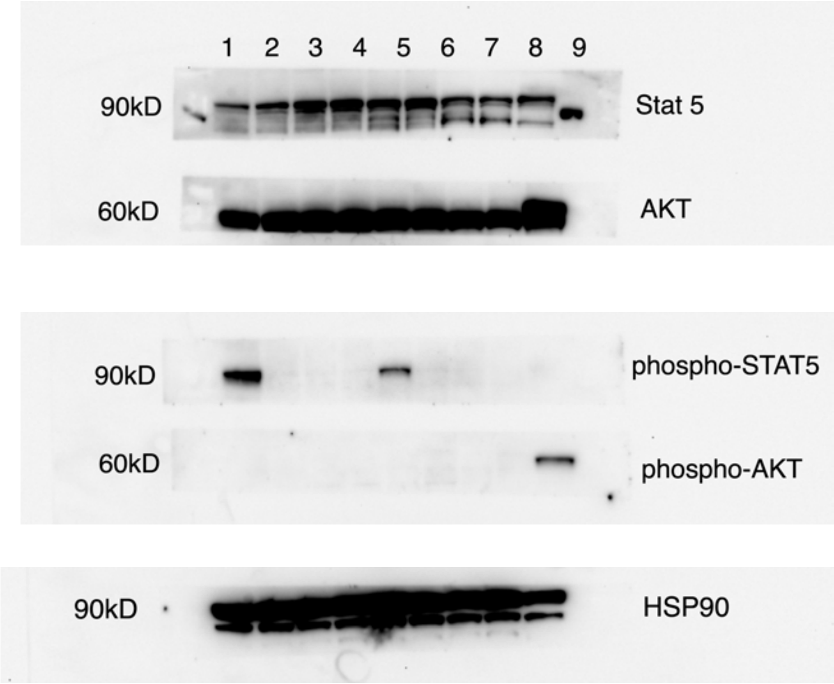

Figure 5- part 2 blot and colorimetric merged

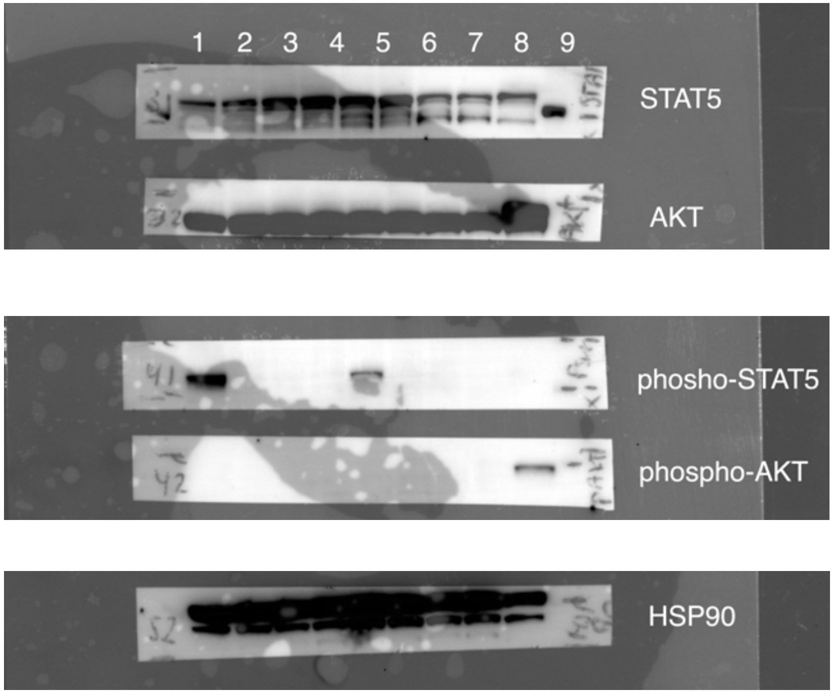

Supplement: S1 Raw images — Images are showed before (called colorimetric) and after exposure (called blot). (PDF) [file pone.0235766.s012.pdf]
